# Porosity Local Analysis (PoLA): A New Approach to Describe the Porous Volume Distribution in Amorphous Carbons

**DOI:** 10.1021/acsomega.5c02479

**Published:** 2025-07-15

**Authors:** Alberto Zoccante, Maddalena D’Amore, Ciro Achille Guido, Alessandro Fortunelli, Giorgio Conter, Leonardo Marchese, Maurizio Cossi

**Affiliations:** † Dipartimento di Scienze e Innovazione Tecnologica (DISIT), 19050 Università del Piemonte Orientale, viale T. Michel 11, I-15121 Alessandria, Italy; ‡ Consiglio Nazionale delle Ricerche, CNR-ICCOM, via Moruzzi 1, I-56124 Pisa, Italy; § Centro di Ricerca e Sviluppo per il Risanamento e la Protezione Ambientale (Centro RiSPA), Joint-Lab DISIT/Syensqo, viale T. Michel 11, I-15121 Alessandria, Italy

## Abstract

A new procedure, named PoLA (Porous Local Analysis), is presented to describe the porosity of amorphous carbons accurately. Unlike models based on predefined geometrical pores, PoLA is based on a point-by-point description of the inner void, and it is particularly suitable for amorphous materials. The porous volume is partitioned into small elements (blocks) of user-defined size, and each block is assigned a micro-, meso-, or macroporous nature according to its minimum distance from the material walls. This method is very fast and characterizes any porous volume uniquely: most importantly, this distribution of volume allows one to predict the gas adsorption behavior of the material. To show this, a number of carbon models have been defined, spanning a large range of porosities, and the adsorption isotherm of nitrogen at 77 K has been accurately simulated with Grand Canonical Monte Carlo in each model. We show that PoLA porous volume distributions and adsorption isotherms are strongly correlated so that N_2_ isotherms at 77 K can be accurately predicted by a machine learning procedure on the basis of PoLA results. We expect that this approach will be of great help in the design of new adsorbents and in the interpretation of experimental gas adsorption.

## Graphical abstract

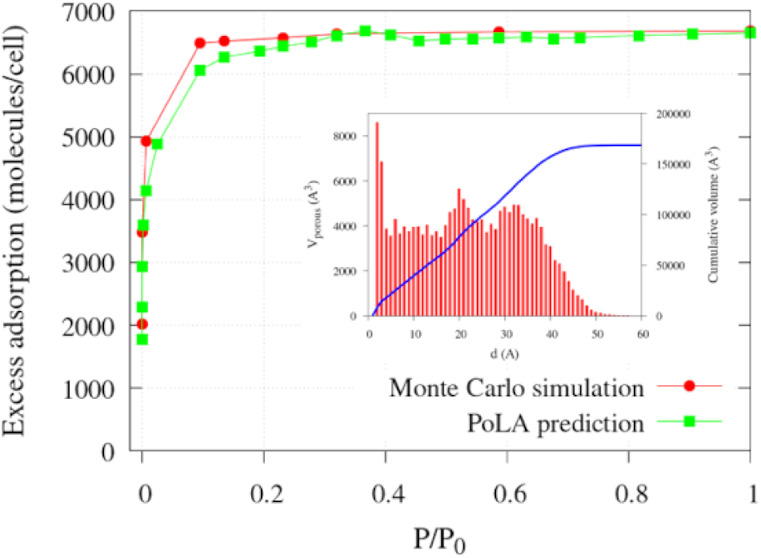

## 1 Introduction

Nanoporous materials are produced and studied with an ever-increasing interest worldwide for their importance in gas storage,^1–4^ gas separation and purification,^5–9^ energy storage,^10–12^ catalysis,^13–19^ and many others.^20–24^ Out of the many classes of such materials (e.g., MOF,^25,26^ zeolites,^27^ porous aromatic frameworks,^28^ hyperreticulated polymers^29^), porous activated carbons are particularly relevant for their high affinity with gases of great practical importance (as H_2_, CH_4_, CO_2_) as well as for many organic compounds to be filtered and extracted from soil and water.^30–32^ Porous carbons display a great morphological and textural variety suited for different applications, and they can be produced in large quantity with relatively inexpensive syntheses, also starting from agricultural or industrial wastes.^33–35^

Most carbon applications are based on the physisorption of small molecules inside their inner, nanosized voids: it is of primary importance, then, that the porous structure is characterized uniquely and possibly correlated to the adsorption patterns. The most used descriptor for material porosity is the pore size distribution (PSD),^36–40^ which can be determined experimentally by adsorbing a probe gas (typically, N_2_ at 77 K, Ar at 87 K, or CO_2_ at 273 K) and fitting the adsorption isotherms with parametrized methods rooted in density functional theory (DFT) for inhomogeneous fluids.^41–44^ Nonlocal, quenched solid, and bidimensional (NLDFT, QSDFT, 2DDFT, respectively) variants of the method have been proposed to represent porous solids with increasing accuracy and reproducibility.^45–51^

DFT models describe the porous structure of the materials as a collection of “pores” of definite shape, typically spheres, cylinders, or parallel slits, fitting the experimental isotherm with the best combination of such cavities: the “pore size” associated with each element (the diameter for spheres and cylinders, and the distance between opposite planes for slits) is combined to provide the PSD. This vastly popular approach became a standard in the scientific community also because it provides a simple and recognizable description of very different systems, allowing comparison of their porous structures. Similar methods are often applied also to atomistic models: for instance, the popular PoreBlazer approach^52–54^ provides a geometrical PSD for theoretical models of porous solids as a collection of overlapping spheres filling the inner voids (see also refs 55–59 for alternative PSD definitions based on geometrical pores). Similarly, Zeo++ method^60,61^ defines a Voronoi network to describe the void space in crystalline materials (but the method can be applied to amorphous systems as well), obtaining the PSD as a collection of spheres fitting the pores.

While in ordered porous solids, like MOF or zeolites, the inner structure can be actually seen as formed by geometrical pores, this model is clearly less suited to amorphous materials such as carbons: trying to describe carbon porosity in terms of spheres or cylinders leads to artifacts, which make the PSD less realistic and predictive for this kind of systems. Such limitation of the standard PSD model for amorphous solids has been already discussed and led sometimes to alternative proposals. For instance, in ref 62, the “one-dimensional” parameter (the pore size) was abandoned in favor of a large collection of 3D carbon structures used to fit the experimental adsorption isotherms with suitable regression models. This approach brings useful information about the possible atomic structure starting from probe adsorption isotherms, but it does not provide any “metrics” to compare different systems and cannot be used to characterize theoretical models unless an adsorption curve is simulated somehow.

In this article, we present a new model to describe the distribution of the void volume inside a porous solid, of which the atomistic structure is known: we will show its application to a series of porous carbon models with different densities and porosities. This model does not rely on pores of predefined shape: the void volume, instead, is analyzed locally and each point is assigned a “minimum distance from opposite walls”, which determines if that point (actually, a small region around it) belongs to the ultramicro-, micro-, meso- or macroporous volume, according to IUPAC definitions.^63^ A detailed output is also provided, in which the whole porous volume is partitioned in several contributions of given size (adjustable by users), producing histograms that resemble the PSD mentioned above, though with different profiles and based on a different model.

This approach is suited to ordered as well as amorphous materials, although it is expected to be particularly useful in the latter case, for the reasons discussed above. In addition to being very flexible and fast, compared to other methods that partition the porous volumes in “pores” of a given shape, the main advantage of the method is that its porous volume distributions are strongly correlated to the gas adsorption isotherms. We will prove this correlation by simulating N_2_ adsorption isotherms in a number of porous models and showing that they can be effectively predicted on the basis of our porous volume distributions. Since the number of models is quite large, in the following, only some examples are illustrated explicitly, while the whole set of results is shown in the Supporting Information (SI).

## 2 Porosity Local Analysis (PoLA)

### 2.1 Basic Concept

Since pores present very different morphologies, the IUPAC classification refers to their “width” without specifying a particular shape, not what width means in general: then, one defines micropores with widths below 20 Å, mesopores between 20 and 50 Å, and macropores above 50 Å (sometimes ultramicropores, below 7 Å, are added).^63^ A more precise definition can be related to the shape of the interaction potential acting on a probe molecule inside the pore, as sketched in Figure 1. In narrow (micro) pores, a gaseous probe interacts strongly with more than one wall, so at least in one direction the interaction potential appears as a single well with a clearly defined minimum (Figure 1a); when walls get further apart, as in mesopores, the potential becomes a double well (Figure 1b), and if the probe interacts with just one solid surface, the potential resembles locally a Morse-like function (Figure 1c).^64^

**Figure 1:**
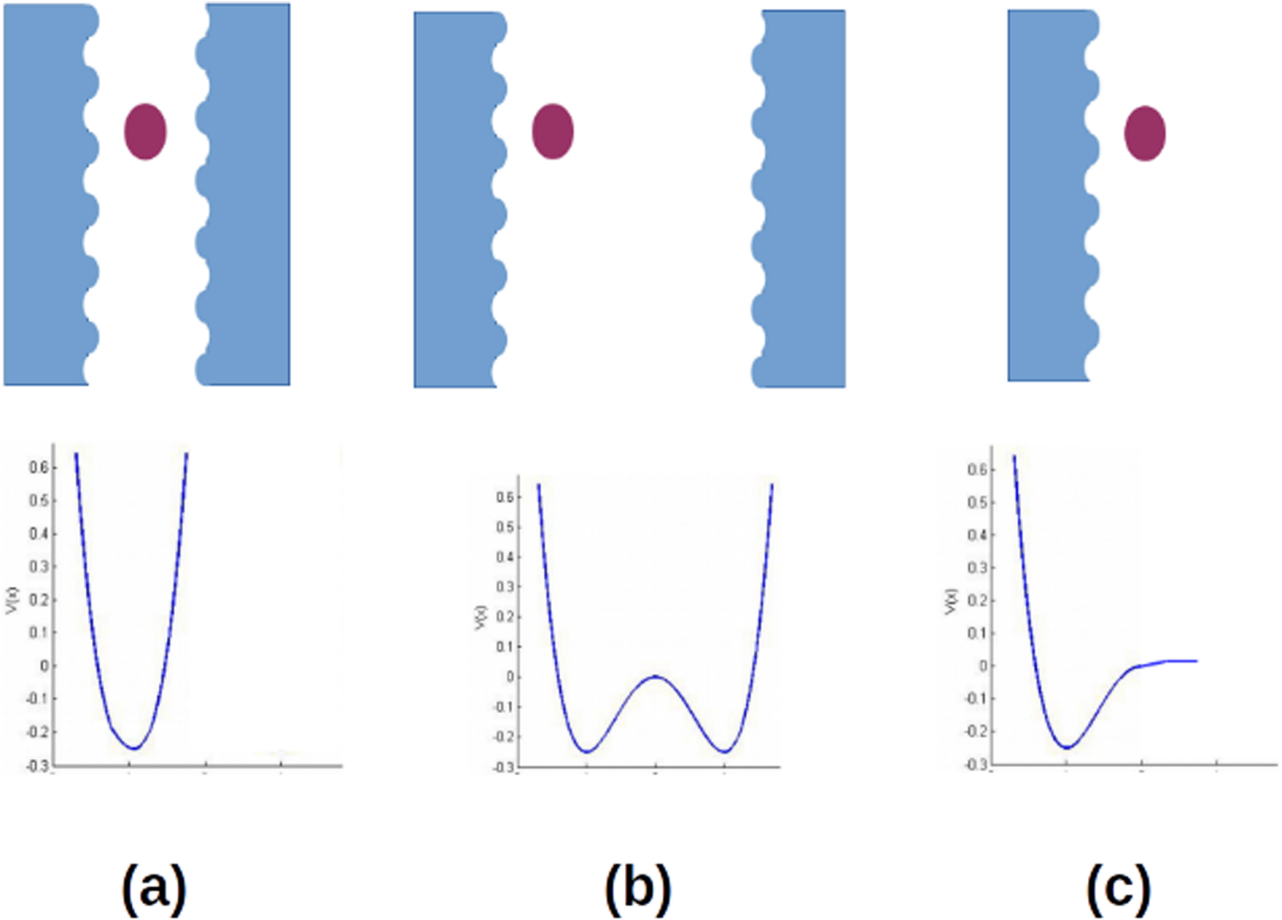
Classification of pores and interaction potentials: (a) micropore, (b) mesopore, and (c) macropore.

Physisorption is obviously driven by the interaction potential experienced by the gas molecules with the solid walls: reasonably, every point (or small region) of the porous volume contributes to the physisorption process according to the local shape of the interaction potential. The model proposed here is based on such a *local* analysis of the porous volume, to assign every region to the suitable class of porosity (hence, of interaction potential), as shown in Figure 2.

**Figure 2:**
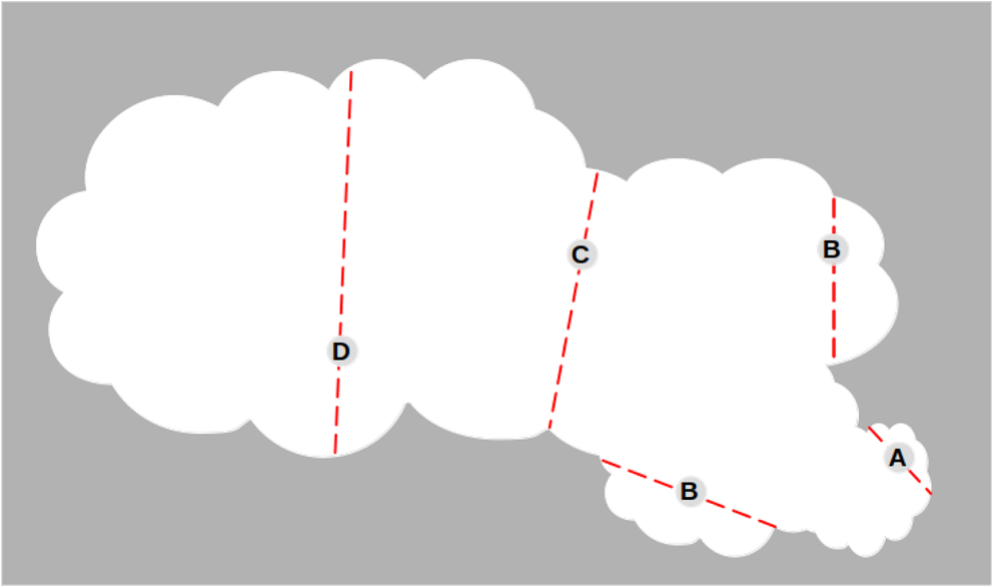
Local analysis of the porosity: a molecule in A would experience a very peaked single well potential, as in a conventional ultramicropore; in B a less steep potential, as in a micropore; in C and D double well potentials, with different intensities, as in small or large mesopores.

In the most commonly used methods based on geometrical pores (e.g., PoreBlazer), the whole volume contained in, for instance, a spherical pore of 15 Å radius is considered microporous, whereas all of the volume in a sphere of 25 Å radius would be mesoporous. In this way, when considering the adsorption behavior, every point within that pore would be actually considered equivalent. Conversely, in the PoLA (Porosity Local Analysis) method, each point, even in a spherical cavity, will be classified according to its geometrical distance from the surrounding material, better reflecting its interaction potential. Schematically, a part of the volume will behave as in ultramicropores, another as in micropores, and regions around the center (for large enough pores) will be recognized as actually mesoporous in nature (see also Figures S1–S3). Clearly, the difference between the models is much more important in amorphous systems, like carbons, where the porous volume is poorly described by spherical (or cylindrical or slit-like) “pores”: in this case, we believe that PoLA may describe the adsorption properties of the system better.

### 2.2 PoLA Algorithm

PoLA analysis is performed by a simple Python3 code with a FORTRAN90 computational core; the input comprises an atomistic model of the solid along with some user-defined parameters mentioned below. The model is defined by atomic numbers and Cartesian coordinates in a periodic orthogonal box, with edges *R*_*x*_, *R*_*y*_, and *R*_*z*_; other periodic cells can be analyzed, provided the atoms are wrapped inside an orthogonal supercell.

The cell is divided in N small cubic regions (blocks) of edge *l* and volume *v* = *l* ^3^ so that $N=N_{x}\times N_{y}\times N_{z},N_{i}=R_{i}/l$ ; the size of *l* is provided as input: in the tests reported below, *l* ranges from 0.25 to 1 Å.

Every block containing one of the solid atoms is defined as filled, as are all of the blocks whose centers fall inside the van der Waals spheres assigned to each atom. The small void regions remaining “trapped” in a filled area, without connections with the other voids, are also filled to avoid the appearance of spurious very small “pores”; the maximum size of such regions, *D*, is provided in the input: in the tests below, *D* ranges from 5 to 10 Å. The blocks that remain void after this process constitute the porous volume to be further analyzed: the above procedure is illustrated in Figure 3.

**Figure 3:**
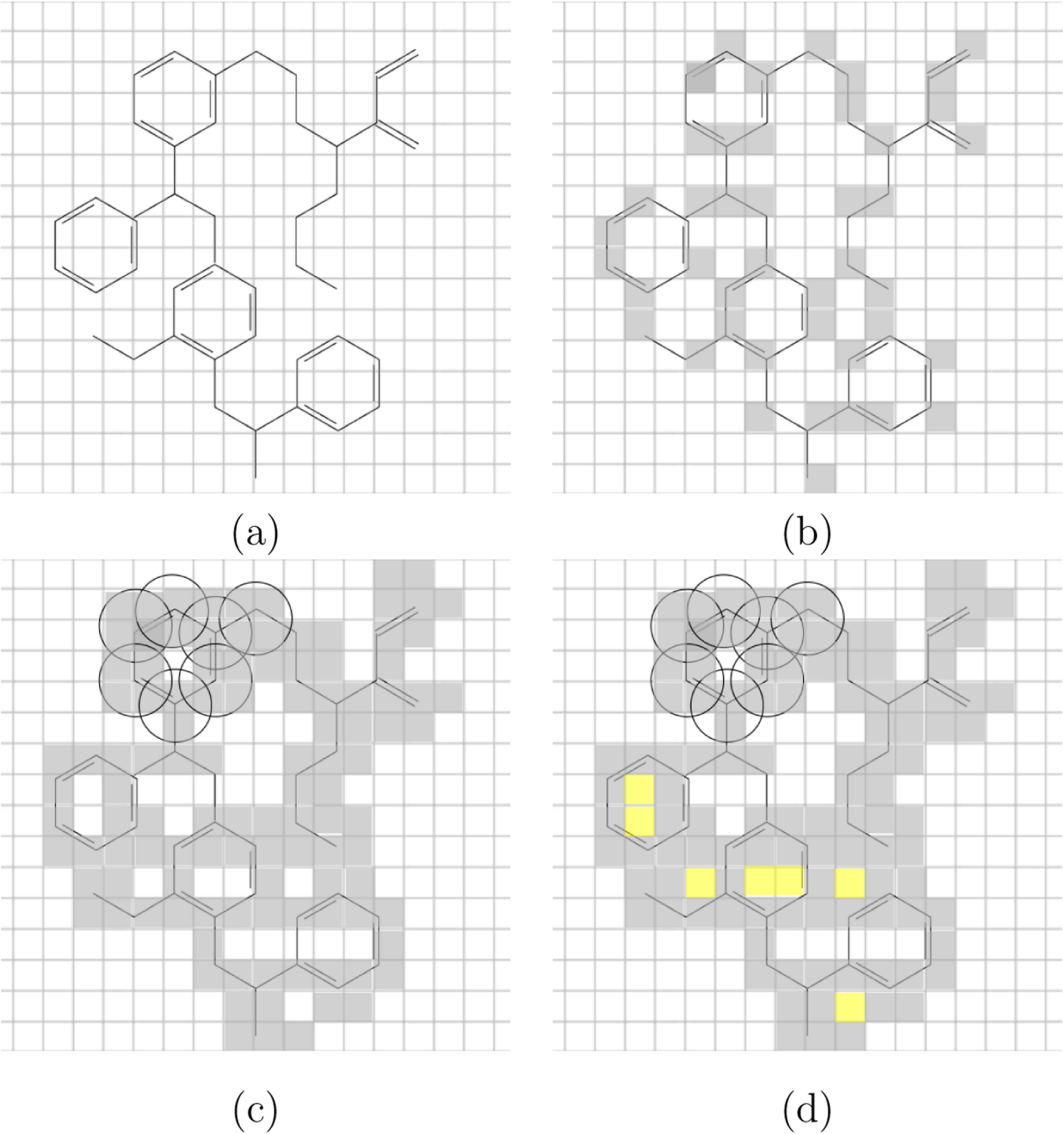
2D sketch of the PoLA definition of the porous volume. White: empty, gray/yellow: filled block; (a) material atoms are immersed in a regular grid; (b) blocks containing an atom are filled; (c) blocks whose center falls inside an atomic van der Waals (vdW) sphere are filled, some of the vdW regions are shown as circles; and (d) void blocks (here in yellow) with too small distances from walls in all directions are filled. The remaining white (void) blocks constitute the porous volume.

For every void block *i*, the code finds the shortest distance from opposite walls: a number of different directions are considered, scanning the spherical angles (38 values of angles are used, scanning actually only one hemispace, for the reason explained below). Starting from the center of block *i*, successive steps of length *l* (as the block edge) are performed along the chosen direction: if the step gets to a void block, the process goes on, and it stops when a filled block is found (i.e., a wall has been touched). Then, the opposite direction is immediately searched (flipping the spherical angles) until another wall is found: the distance between the filled blocks is computed and stored. When all of the directions have been scanned, the minimum distance between opposite walls *d*_*min*,*i*_ is selected and attributed to the *i*th block: the procedure is sketched in Figure 4.

**Figure 4:**
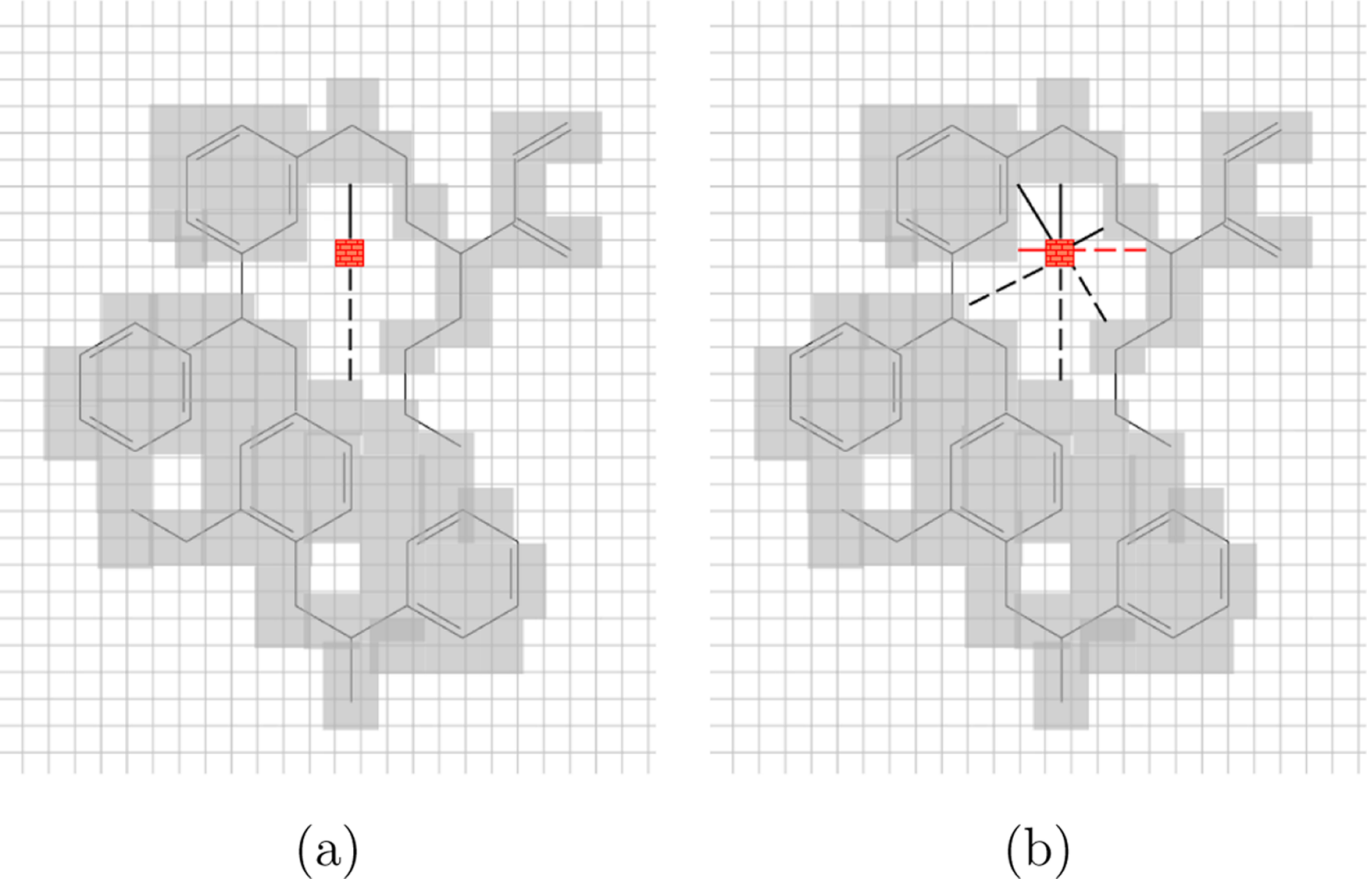
Definition of the minimum distance from opposite walls for a void block (with red pattern); (a) one direction (black continuous line) is searched, and then the opposite (dotted line) until filled blocks are found and (b) many directions are scanned, and the shortest distance (red line) is selected.

After checking all of the void blocks, the porous volume distribution, PVD­(*d*), is obtained as a function of distance *d* by summing the volumes *v*_*i*_ for *d* – δ/2 ≤ *d*_*min*,*i*_ < *d* + δ/2. The most detailed PVD is achieved with δ = *l*, while a more concise description of the porous volume can be obtained for instance by adding the volume of all of the blocks with *d*_*min*_ < 20 Å, 20 Å ≤ *d*_*min*_ < 50 Å, and *d*_*min*_ ≥ 50 Å (corresponding to conventional micro-, meso-, and macroporous volumes); other partitions can be easily defined in PoLA.

Along with *d*_*min*,*i*_, also the maximum distance between opposite walls, *d*_*max*,*i*_, is registered for all of the void blocks: it is used only to identify the spurious empty regions that are not to be included in the porous volume, as said above. Block *i* is filled if *d*_*max*,*i*_ < *D*, i.e., than the threshold defined in the PoLA input.

## 3 Other Methods

### 3.1 Definition of Carbon Models

To test PoLA performance and verify to what extent the porous volume distributions are correlated to the adsorption isotherms, we prepared a data set of 41 carbon models, all comprising the same atom types, but spanning a large range of porosities. Most of the models (1–33) were prepared by the following procedure, which provides micro- as well as mesoporous systems easily. A cubic box of 60 Å edge is divided into 1 Å^3^ small cubic blocks: at the beginning, all of the blocks are “filled”, and the user decides the final porous volume and density. Then, starting from a randomly chosen block, a cavity is dug by emptying a rectangular cuboid centered on the selected block (the length of the edges is randomized and selected from a log-normal distribution with user-defined parameters), and the procedure is iterated either enlarging the current cavity (i.e., moving to one of the just emptied blocks) or jumping to a filled block to begin a new cavity, depending on a randomized draw. This procedure can be tuned to generate widely distinct porosities from samples dominated by micropores to models with meso- and macroporous cavities.

When the desired total porous volume is reached, the portion of the box that has not been hollowed is filled by a suitable number of corannulene (C_20_H_10_) moieties in random positions and orientations, avoiding too close contacts, until the required densities are reached: this step is performed with the Packmol package.^65^ Corannulene (an aromatic, bowl--shaped molecule) has already been used to model carbon structures in cases when a realistic bond network is not required.^66,67^

Three more models with a cubic cell of 50 Å edge, numbered 34–36, and one with 85.5 Å edge (model #37) were prepared via a different procedure, based on the DynReaxMas approach.^68,69^ DynReaxMas is based on a reactive molecular dynamics (R-MD) method, with the C-2013 ReaxFF force field;^70,71^ this method includes the massaging of the potential energy surface (PES) to accelerate the simulation of dynamical processes and finally produce the carbon models,^68^ in this case complemented with an additional step of thermal curing to get rid of the highest energy defects.^69^ More details on the procedure are provided in the SI.

Furthermore, four carbon models were taken from the data set prepared in ref 62: as already commented above, in that work, a large set of models was used to develop the so-called 3D-Vis method to characterize the porous structure of a sample from its N_2_ adsorption isotherm. The Cartesian structures of all of these models, along with the corresponding nitrogen adsorption isotherms simulated at 77 K with Grand Canonical Monte Carlo, are provided in the Supporting Information of ref 62. Out of this data set, we chose models #2, #31, #58, and #75 (which became our models 38–41, respectively) as they offer a large variety of porous volume distribution.

Models 1–36 were used to train and test the machine learning algorithm looking for the porosity/adsorption correlation; models 37–41 were not included in the training set but rather used as an external validation set.

A graphical representation of the unit cell for three carbon models (one for each preparation method, as described above) is presented in Figure 5; pictures of all of the models, along with their Cartesian coordinates, are provided in the SI.

**Figure 5:**
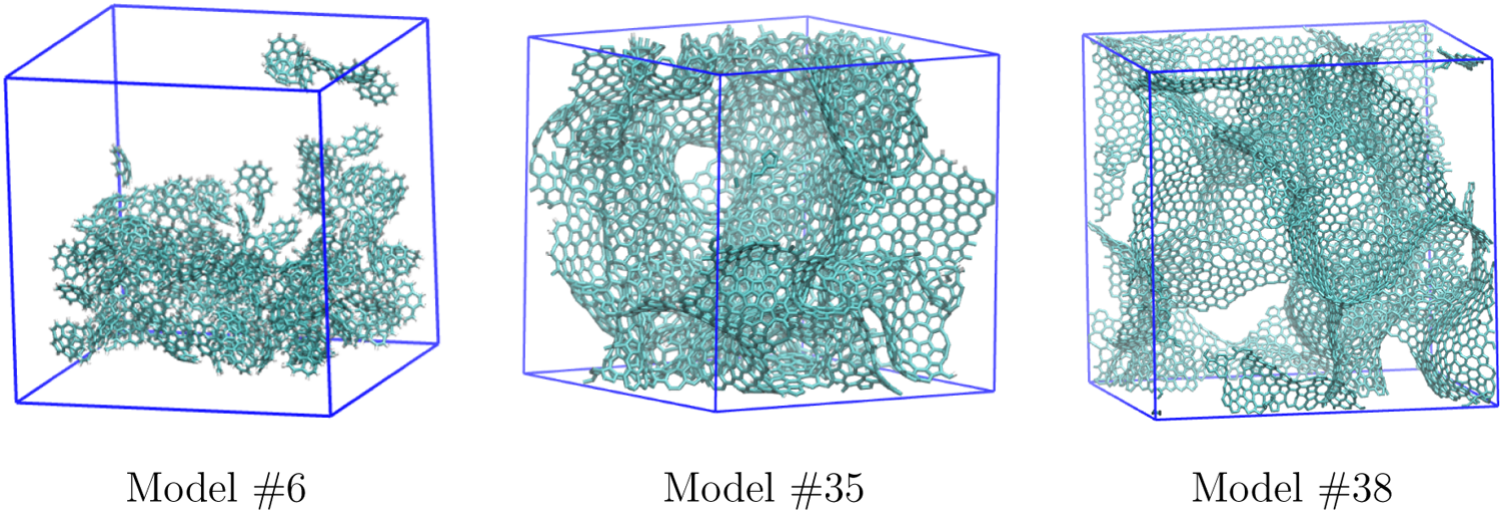
Picture of the unit cell for some representative carbon models: #6 prepared with the procedure described in Section 3.1; #35 prepared with DynReaxMas (see above); and #38 from the carbon coordinates provided in ref 62 (see text).

### 3.2 Monte Carlo Simulation of Adsorption Isotherms

For all of the carbon models described above, the adsorption isotherms of N_2_ at 77 K were simulated by the Grand Canonical Monte Carlo (GCMC) method as implemented in the Cassandra code,^72^ except for models 38–41, for which the isotherms were taken from the literature as explained above. The carbon framework geometry as well as the gas interatomic distance were kept fixed during the simulations so that the force field accounted for nonbonded interactions only. Nitrogen molecules were simulated with a three-body model, with a partial charge in the mid of the interatomic bond (set to 1.10 Å length); Lennard-Jones (LJ) interaction parameters and partial charges were taken from refs 73–75 . The interactions with the carbon framework were simulated with the LJ parameters and charges proposed by Di Biase et al. and then largely used in the literature^76–78^ all of the force-field parameters are listed in the SI.

Chemical potentials were correlated to gas pressures by simulating with Cassandra pure nitrogen densities at 77 K and comparing the values with the very accurate state functions reported in the NIST Website.^79^

Then, the adsorption was simulated for 24 values of pressure (from 3.70 × 10^–5^ to 9.99 × 10^–1^ bar, the latter being very close to N_2_ condensation pressure at this temperature): all simulations comprised 10^6^ equilibration and 5 × 10^6^ production steps. The number of adsorbed molecules, *N*_*MC*_, was averaged over the last 10^6^ steps of the production run. This procedure led to smooth and stable adsorption curves for all of the pressures in the models described in Section 3.1, with the exception of the larger carbon model #37 in the validation set: in this case, much longer runs were necessary to achieve converged adsorption at high pressures; so, for this model, the GCMC process was extended to 14 × 10^6^ production steps.

For consistency with the experimental practice, we considered the excess adsorption isotherms, defined as *N*_*exc*_ = *N*_*MC*_ – ρ_*N*_2__ × *V*_*por*_, where ρ_*N*_2__ is the density of the free gas at the considered pressure, and *V*_*por*_ the total porous volume obtained by PoLA.

All of the simulated N_2_ adsorption isotherms used in this work are provided in the SI.

### 3.3 Machine Learning Prediction of N_2_ Isotherms

To evaluate the predictive capacity of PoLA analysis, we correlated the simulated N_2_ isotherms in the carbon model data set with the corresponding porous volume distributions. Such a task can be ideally tackled by machine learning (ML) algorithms, which have gained a great popularity in recent years for this kind of multiple regression problems, even in applications strongly related to the chemistry of materials.^80–83^ Among the various ML approaches, we resorted to the random forest (RF) procedure, as implemented in the scikit-learn package:^84^ RF offers a high accuracy even with noisy data and avoids any parametric assumptions between input and predicted results, so the possible correlation is likely to be transferable to systems outside the training set, provided the physical interactions are the same. On the other hand, RF tends to be more computationally demanding than other ML regressors, but in our case, the data set is quite limited, so the computational cost is not an issue: indeed, all of the regressions described in the following were carried out on a laptop with wall times from few seconds to 1 min.

The RF training was performed using 1000 estimators; the target values were the excess adsorptions (expressed as the number of N_2_ molecules in the model unit cell) computed with GCMC for the various pressures; a single regression process was performed for all of the pressures in the whole training set. Different choices are possible for the input data (“features”), i.e., the geometrical elements to be correlated to adsorptions. PoLA provides PVD­(*d*) as well as cumulative volumes (see Figure 6a and b, respectively) and both quantities can be used in the training; since RF is known to work better by comparing *relative* feature distributions, PVD and cumulative volumes can be scaled by the sample total porous volume, so all of the systems are described on the same scale. Using scaled quantities implies that the predicted isotherm has to be multiplied by the corresponding total porous volume before being compared to the GCMC counterparts.

**Figure 6:**
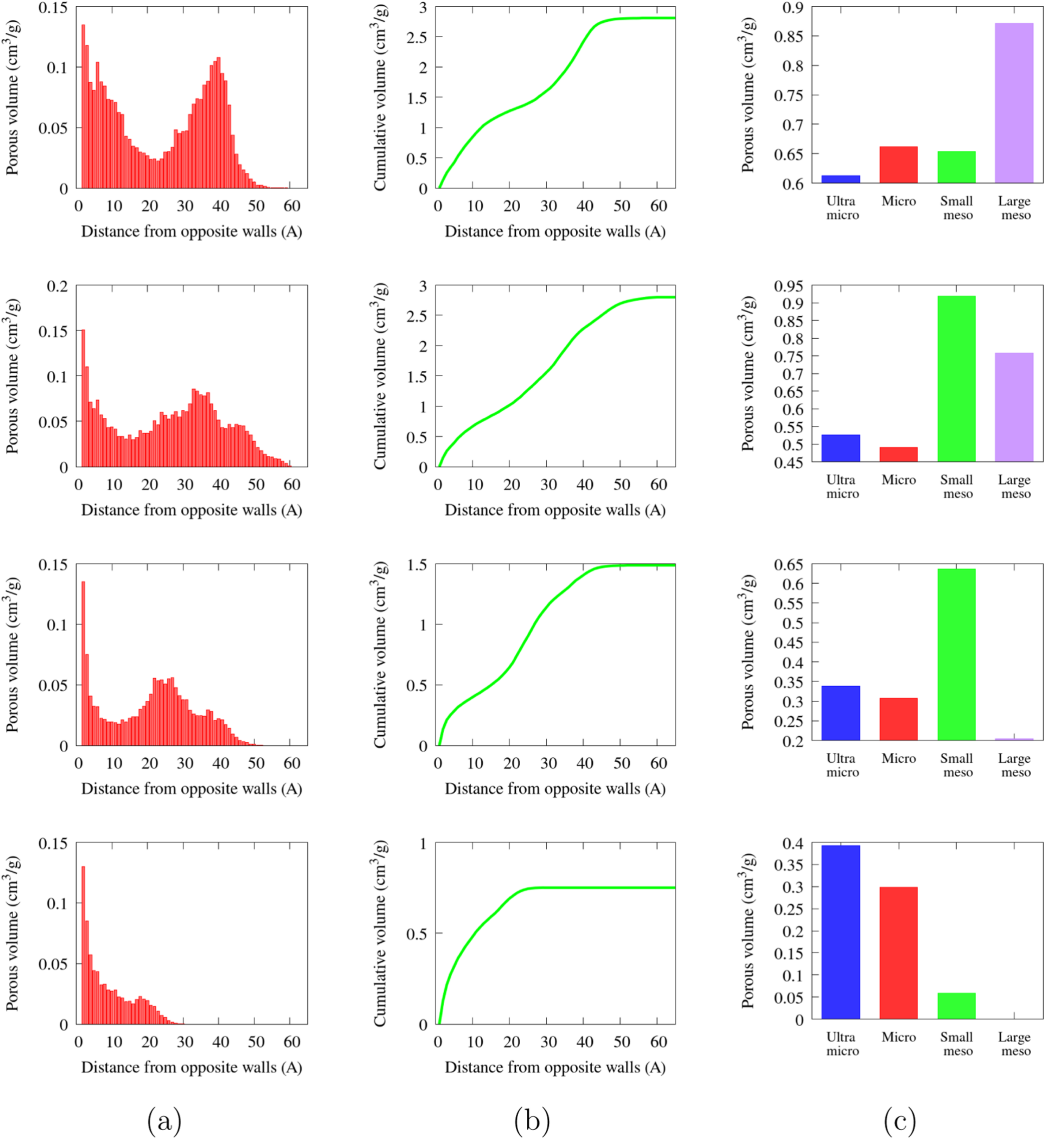
Results from PoLA analysis on some carbon models (the complete set is reported in the SI). From top to bottom, models #6, #10, #17, and #31; (a) porous volume distribution, on abscissa the values of the distance between opposite walls, on ordinate the total volume of the void blocks with that value of *d*_*min*_; (b) cumulative porous volume, i.e. the cumulative sum of the values in the first row; and (c) example of more concise output, i.e., the sum of the block volumes for *d*_*min*_ < 7 Å (ultramicroporous volume), 7 Å < *d*_*min*_ < 20 Å (microporous), 20 Å < *d*_*min*_ < 35 Å (small mesoporous), and 35 Å < *d*_*min*_ < 50 Å (large mesoporous).

The performance of these various strategies can be evaluated using the mean and the maximum absolute errors for all of the predicted isotherms (for each model, the GCMC and predicted adsorptions can be compared for every pressure value). The results are commented below (Section 4.2) and illustrated in the SI: we anticipate that the best performance was obtained using as features the cumulative volumes scaled by the total volume.

## 4 Results and Discussion

### 4.1 Porous Volume Distributions

All of the carbon models were analyzed with PoLA to obtain their porous volume distributions. The procedure proved very efficient and fast: with a single processor Intel i7, using *l* = 1 Å, i.e., dividing the model boxes in blocks of 1 Å^3^ volume, every model was analyzed in few seconds. Increasing the accuracy by putting *l* = 0.25 Å, that is multiplying by 64 the number of blocks, the longest wall time was about 28 min (note that passing from *l* = 1 Å to 0.25 Å, the total porous volume computed by PoLA changed at most by 1.5%; see below for a more detailed analysis).

In Figure 6 and Table 1, the results of PoLA analysis (*l* = 1 Å, threshold for removing the small pores *d*_*max*_ = 7 Å) are illustrated for four representative models with different densities: the analysis of the complete data set is reported in the SI.

**Table 1:** Textural Properties of Some Carbon Models Analyzed with PoLA^a^

			microporous vol.		mesoporous vol.	
model	density	total porous volume	ultra	super	small	large
#6	0.300	2.806	1.274		1.524	
			0.613	0.662	0.654	0.871
#10	0.300	2.798	1.017		1.678	
			0.526	0.491	0.919	0.758
#17	0.500	1.486	0.645		0.840	
			0.338	0.307	0.636	0.204
#31	0.798	0.751	0.692		0.059	
			0.393	0.299	0.059	0.000

a Density in g/cm^3^, specific volumes in cm^3^/g; the classification of porous volume is based on the following thresholds: ultramicro, distance from opposite walls below 7 Å; supermicro, below 20 Å; small meso, below 35 Å, and large meso, below 50 Å (larger distances from walls are associated to macroporous volume, not present in these models, except in model 10, where it is 0.103 cm^3^/g).

PoLA characterized finely all of the models: in Figure 6, the four samples present very different profiles, also because they have dissimilar structures and densities, but looking at the complete results listed in the SI, we see that carbons with the same density also exhibit distinct volume distribution profiles, even though the total volumes may be similar. On the other hand, structures obtained with very different approaches, as described in Section 3.1, and with different morphologies, are described with the same efficiency.

Thanks to the local analysis it performs, PoLA can describe each structure in greater detail and accuracy than models based on pores with predefined, geometrical shapes. The very concept of “pore”, actually, may not be fully suited to amorphous materials like porous carbons, which can hardly be seen as a collection of distinct pores (unlike crystalline systems like MOF or zeolites, to a certain extent), but rather as a nonhomogeneous distribution of voids delimited by irregular walls.

It is instructive to compare the distribution of the porous volume proposed by PoLA and other well-known methods commonly applied to characterize amorphous porous solids. For instance, in Figure 7, we compare PoLA distribution with PoreBlazer, probably the most widely used tool, which describes the voids inside the material with a collection of overlapping spheres, whose diameters are assigned as pore sizes. Not surprisingly, the two methods provide quite different profiles for the porous volume distribution, though the total volumes agree very well, as evidenced in Figure 7 (here, we are using the so-called geometrical volume provided by PoreBlazer, obtained with a point-like probe). In general, PoLA distribution contains systematically larger amounts of micro- (and also ultramicro) volume, meaning that stronger adsorbate/material interactions are predicted, at least in a part of the void space.

**Figure 7:**
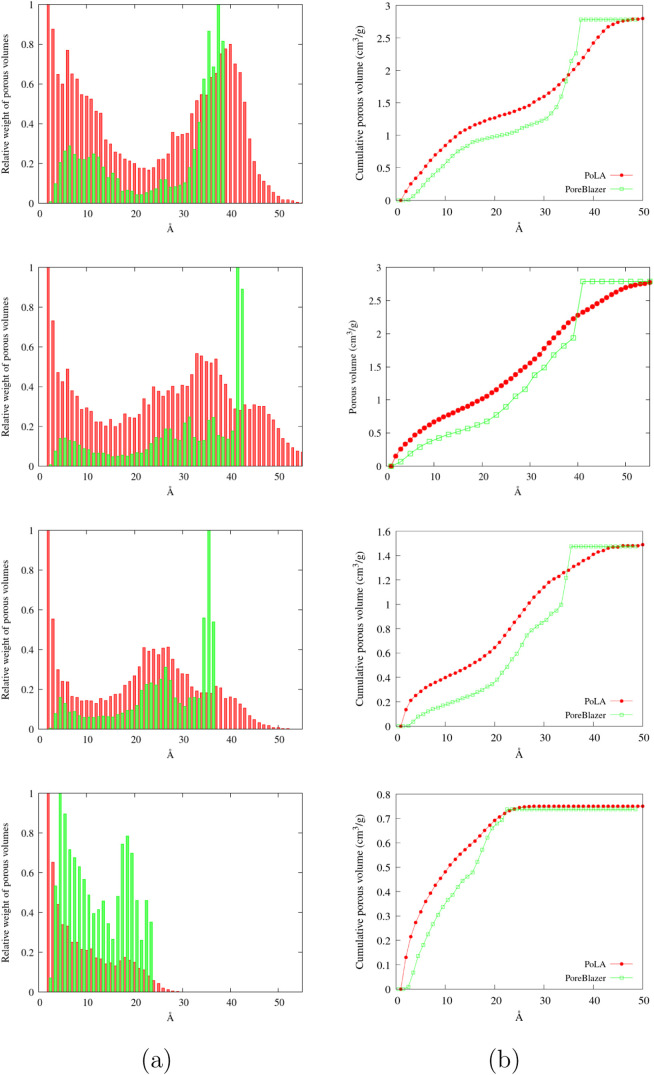
Comparison between the porous volume analysis provided by PoLA (red) and PoreBlazer­(green). From top to bottom, models #6, #10, #17, and #31; (a) on the abscissa, the distance between opposite walls for PoLA and the pore diameter for PoreBlazer, on ordinate the relative weight of the contributions to the total porous volume and (b) cumulative porous volume as a function of the wall distance/pore diameter.

This is not surprising either: not all of the volume elements inside a sphere have the same “minimum distance from opposite walls”; actually, the closer a point is to the surface, the shorter is the sphere chord passing through that point, and this is the quantity used by PoLA to classify the corresponding volume element. So, if we had a spherical cavity with diameter 20 Å, a model based on geometrical pores would report a single pore of volume $\frac{4}{3}\pi 10^{3}\;{Å}^{3}$ at 20 Å, while PoLA would return a wide distribution of “block volumes” with distances from 2 *l* to 20 Å, with the same total volume. This feature is graphically shown in the SI, where we also show how PoLA discriminates between pores with different shapes, as, for instance, slit and spherical pores with the same “size”, where models based on geometrical cavities would return the same pore size distribution.

Analogous conclusions can be drawn from the comparison between PoLA and other methods commonly used to describe porous volume distributions, namely, Zeo++ ^60,61^ and 3d-Vis,^62^ as illustrated in the SI for the same four models shown in Figure 7. 3D-Vis pore size distributions are similar to PoreBlazer profiles, but in the former method, they come from a mixture of several carbon models used to fit the N_2_ adsorption isotherm in the sample of interest, so the distributions do not match, though the major components are consistent; Zeo++ distributions are quite different from the others because of the peculiar method adopted in this procedure to partition the void space; in addition, Zeo++ provides the volume accessible to finite size probes only, while the other methods allow for point-like probes also, more comparable to PoLA description.

As noted above, the PoLA accuracy is expected to depend on the size of the blocks used to discretize the void volume. To check the weight of this parameter and find a good compromise between accuracy and efficiency, PoLA analysis was repeated on the same samples illustrated in Figure 6 with *l* = 0.5 and 0.25 Å, with the results shown in Figure 8 (for the whole test set, see the SI). Indeed, PoLA output resulted very stable with respect to the reduction of the block size, and even with *l* = 1 Å, that could be considered quite a rough discretization mesh, the cumulative pore volume is almost undistinguishable from *l* = 0.25 Å. As a consequence, *l* = 1 Å was set as the default value for the block size, and it was used in all of the following test calculations.

**Figure 8:**
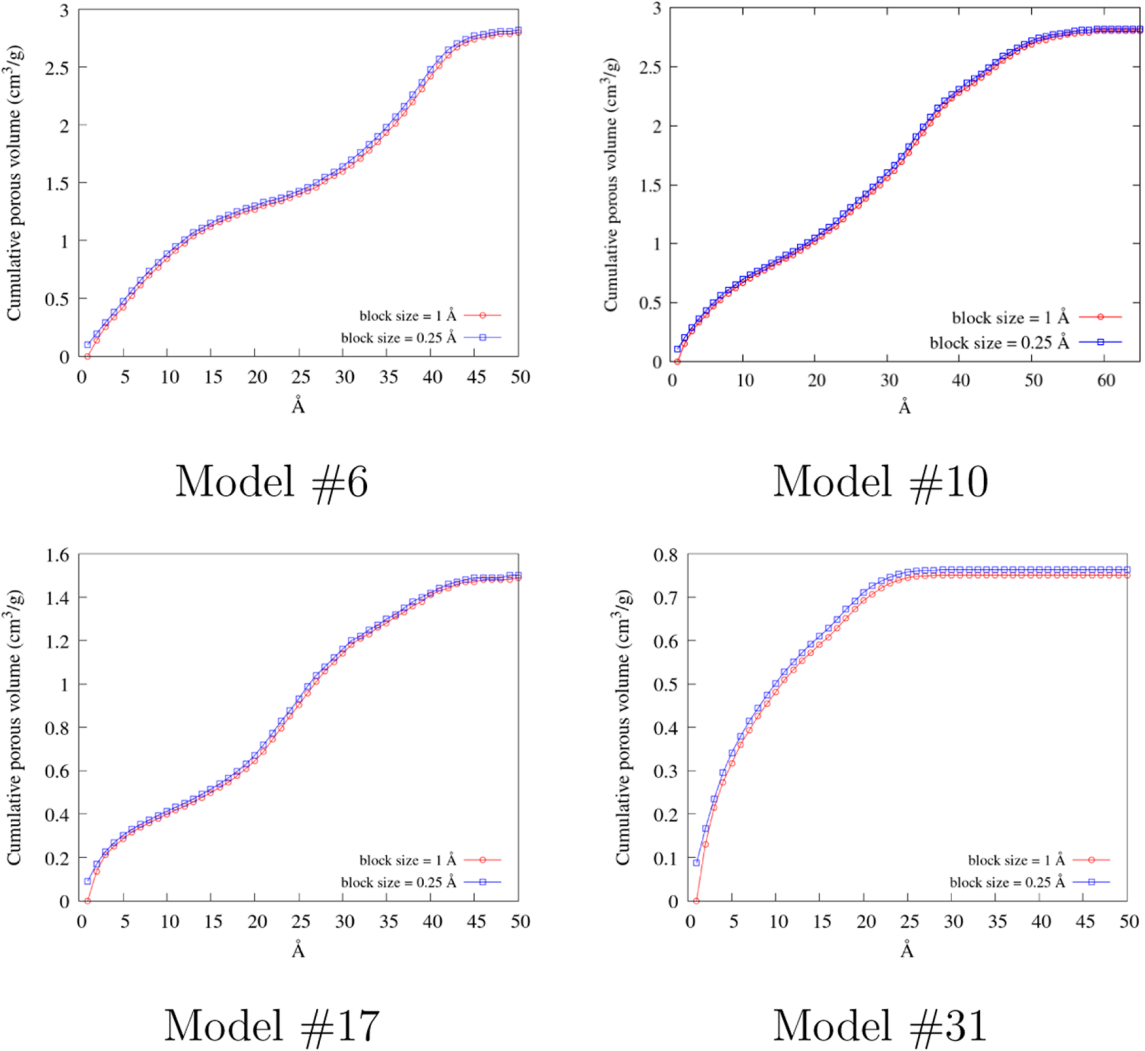
Cumulative porous volume computed with different block sizes: for clarity, only the results for *l* = 1 Å (red circles) and *l* = 0.25 Å (blue squares) are shown.

### 4.2 Prediction of N_2_ Adsorption Isotherms at 77 K

One of the main advantages of the PoLA method, in our opinion, lies in the possibility to predict the adsorption isotherms for various gases on the basis of the porous volume distribution. The physical correlation between porosity and adsorption patterns is obvious: in a homologous series of adsorbents (as for instance considering nitrogen in different unfunctionalized carbons), the isotherm in a particular sample must depend mostly on the distribution of the porous volume in that adsorbent (since different porosities generate different local interaction potentials, as described in Section 2.1). As discussed above, PoLA describes the porous volume distribution in amorphous systems accurately and in detail and we expect that it is particularly suited to establish such a correlation.

To verify this point, we simulated the adsorption of N_2_ at 77 K in carbon models #1–#37 with the GCMC method implemented in Cassandra, while for models #38–#41, the isotherms were taken from the literature, as explained above (all of the isotherms are reported in the SI).

Models #1–#36, along with the corresponding adsorption isotherms, constitute our reference data set: out of the reference set, 8 models (numbered 3, 10, 15, 17, 20, 24, 33, and 36) were selected randomly to form the test group, with the constraint that at least one system for each density was included (see Table S2); the remaining 28 models formed the training set.

Then, the random forest machine learning algorithm was instructed with the training set and applied to predict the excess adsorption isotherms of the models in the test set on the basis of the corresponding porous volume distributions. The results are very satisfactory, as shown in Figure 9: for all of the test models, the predicted isotherms are very close to the GCMC simulations, proving the strong correlation between PoLA textural properties and the adsorption behavior.

**Figure 9:**
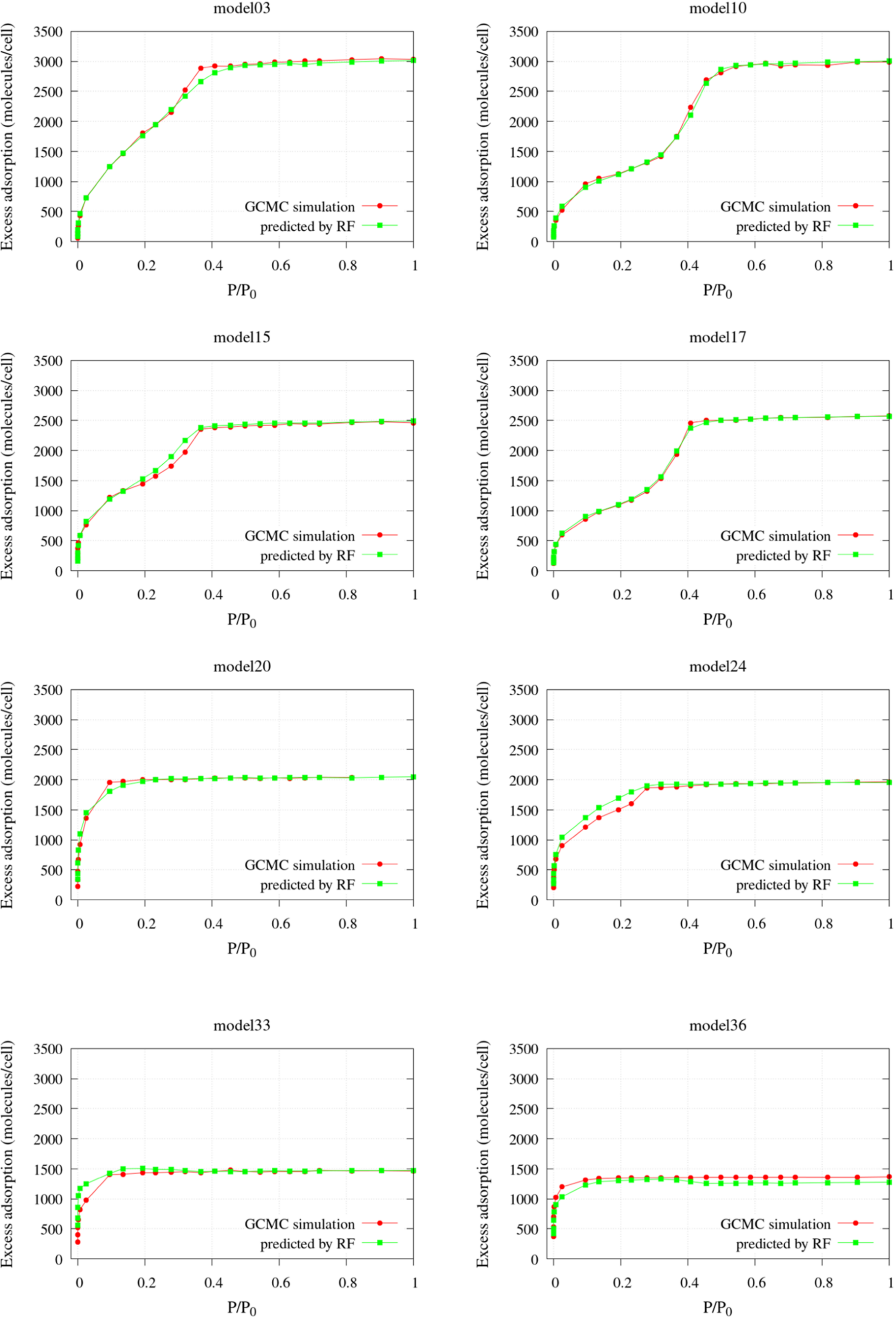
Predicted vs GCMC N_2_ excess adsorption isotherms at 77 K for the test models.

To ensure that this good performance does not depend on the choice of the test set, we performed a complete analysis on the whole reference set: in turn, each system was extracted out of the 36 carbon samples and used as a test, while the remaining samples formed the training set. Then, the trained RF was used to predict the adsorption in the trial sample so that 36 test comparisons were obtained. All of the results are reported in the SI: also in this case, the agreement between predicted and GCMC isotherms is very good, confirming the reliability of the regression based on PoLA volume distributions. The shape of all of the isotherms is reproduced fairly well, also in the case of multimodal adsorptions, so that the contributions from micro- and mesoporosity can be distinguished, and the total amount of adsorbed molecules is predicted accurately as well. Even when the limit of adsorption at *P*/*P*_0_ = 1 does not agree completely, the discrepancies are always below 5% but in most cases the agreement is much better.

It is worth noting that the RF regression was optimized on the given values of pressure, until the saturation condition and the quality of the prediction was evaluated on the whole isotherms: no particular attention was paid to the low-pressure regions, leading to some discrepancies between predicted and simulated isotherms for the lowest loadings. Since the low-pressure branches of the adsorption isotherms are particularly important for some experimental information, further work will concentrate on the prediction in these regions by simulating the adsorption for a large number of low pressures and looking for the correlation with PoLA volumes.

The transferability of the regression parameters to a larger system was tested by using models 1–36 to train the RF algorithm and applying the parameters to the carbon model #37, with a 85.5 Å edge unit cell, described in Section 3.1. The GCMC isotherm in this model was predicted very well too: as shown in Figure 10, both the number of gas molecules adsorbed at a high pressure and the shape of the isotherm are well-described by the RF algorithm trained on PoLA results. It is worth noting that the GCMC isotherm for this large model required a heavy computational effort, as noted above in Section 3.2, and the possibility to *predict* the adsorption curve accurately with minimal cost, once the ML algorithm has been trained, is clearly very promising.

**Figure 10:**
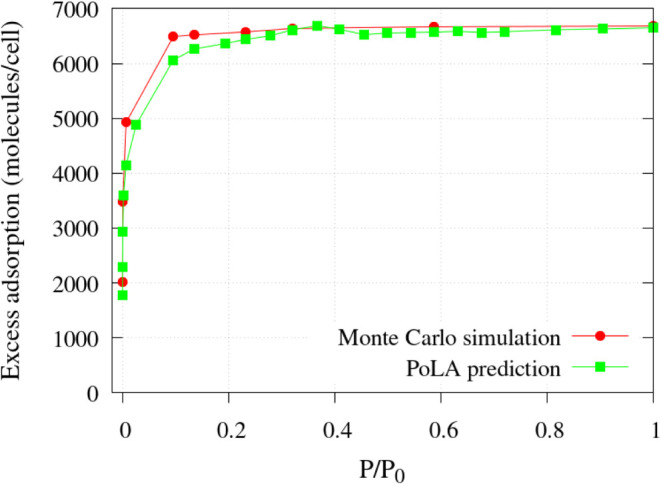
N_2_ excess adsorption isotherm at 77 K in the carbon model with an 85.5 Å cubic edge (Section 3.1) predicted by PoLA and computed by GCMC.

A further test of transferability was performed with models #38–#41, which were prepared with a completely different procedure and present a different morphology (as evidenced in Figure 5 and in the pictures reported in the SI). Also in this case, the RF algorithm was trained with the 36 models of the reference set and used to predict the isotherms, with the results shown in Figure 11: the agreement is again very satisfactory, confirming the reliability and the robustness of the correlation between PoLA volume distributions and N_2_ adsorption at 77 K.

**Figure 11:**
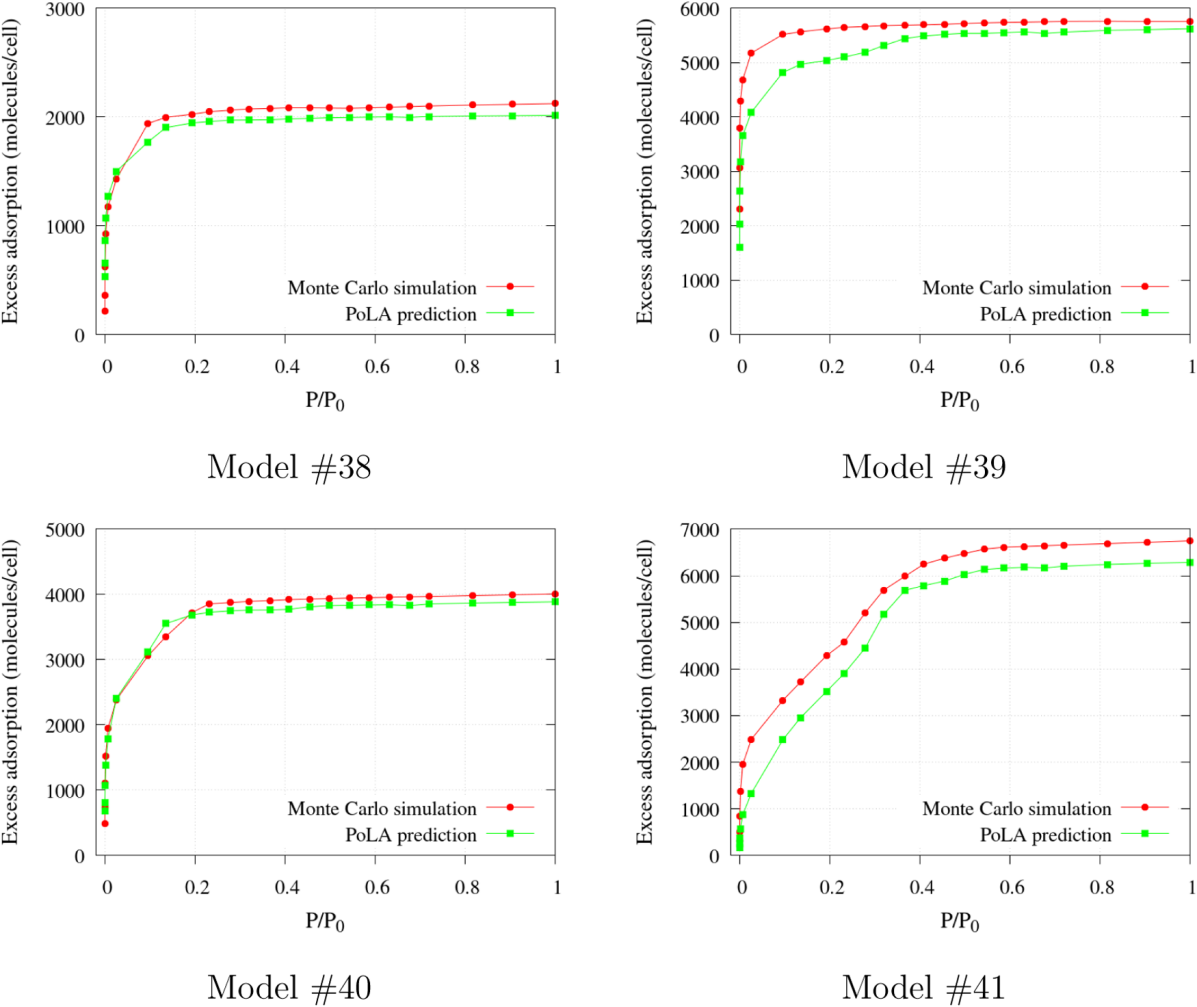
N_2_ excess adsorption isotherm at 77 K in carbon models taken from ref 62, predicted by PoLA and computed by GCMC.

Even more interesting is the chance to correlate the porous volume distribution with the adsorption of other gases too, by training the ML algorithm with the suitable isotherms, with the aim to predict the performance of porous carbons toward different gases once their PVD has been deduced from the measured N_2_ adsorption isotherm, as will be described in a further work.

As noted in Section 3.3, we used cumulative volumes scaled by the total volume as features in all of the regressions reported above (this means that each predicted isotherm was multiplied by the sample porous volume, as noted above). The other possible choices (PVD scaled by total volumes or unscaled PVD or cumulative volumes) provided larger mean and absolute errors when the predicted isotherms were compared to the reference values: the details are reported in the SI.

## 5 Conclusions

We presented an innovative procedure to characterize the inner void in nanoporous solids. The procedure, called Porosity Local Analysis (PoLA), is not based on predefined pores of regular shape, unlike other approaches largely adopted to describe porous solids: instead, PoLA considers each point inside the void and assigns the local (micro, meso, or macro) porosity according to the minimum distance from the material walls.

This method is, in our opinion, particularly suited to characterize amorphous systems like porous carbons: the porosity is classified according to the interaction potential that an adsorbate molecule would feel in each point, depending on the shortest distance from opposite walls. We expect that the resulting porous volume distribution will be strongly correlated to the adsorption isotherms.

This point has been tested by preparing a large number of carbon models, computing their N_2_ adsorption isotherms by the GCMC method, and using a random forest machine learning algorithm to correlate the isotherms to the porous volume distributions provided by PoLA. An excellent correlation was found, proving that N_2_ adsorption isotherms in the model set can be effectively predicted by the RF algorithm on the basis of PoLA porous volumes; the same regression parameters could predict the N_2_ isotherms in a model with a much larger unit cell (model #37) and in some models prepared with a different procedure and with different morphologies (models #38–#41).

On this basis, we believe that PoLA can lead to a substantial improvement in the design and characterization of effective adsorbents, in particular, amorphous porous carbons.

## Supplementary Material





## Notes

PoLA analysis of regular geometric pores; details about the DynReaxMas procedure; parameters of the force field; PoLA analysis of the whole carbon model data set; comparison of porous volumes with different methods (Poreblazer, 3D-Vis, Zeo++); comparison of PoLA volumes with different block sizes; mean and maximum errors for different choices of random forest features; and prediction of N2 adsorption isotherms in the model data set (PDF)
Archives with carbon models coordinates, PoLA analysis of the carbon models, VMD images, and GCMC simulated adsorption isotherms (ZIP)
