# Supplementary material for: Porosity Local Analysis (PoLA): A New Approach to Describe the Porous Volume Distribution in Amorphous Carbons
Source: ACS Omega. 2025 Jul 15;10(29):31623–37. doi: 10.1021/acsomega.5c02479 (PMC12311689; doi:10.1021/acsomega.5c02479)
Supplement: Supplementary file 1 [file ao5c02479_si_001.pdf]

Porosity Local Analysis (PoLA): a new approach to describe the porous volume distribution in amorphous carbons

Alberto Zoccante,[†,¶] Maddalena D'Amore,[†,¶] Ciro Achille Guido,[†,¶] Alessandro Fortunelli,[‡] Giorgio Conter,[‡] Leonardo Marchese,[†,¶] and Maurizio Cossi[*,†,¶]

†Università del Piemonte Orientale, Dipartimento di Scienze e Innovazione Tecnologica (DISIT), viale T. Michel 11, I-15121, Alessandria, Italy

‡Consiglio Nazionale delle Ricerche, CNR-ICCOM, via Moruzzi 1, I-56124, Pisa, Italy

¶Centro di Ricerca e Sviluppo per il Risanamento e la Protezione Ambientale (Centro RiSPA), Joint-Lab DISIT/Syensqo, viale T. Michel 11, I-15121, Alessandria, Italy

E-mail: maurizio.cossi@uniupo.it

Supporting Information

Content

Detailed analysis of regular pores …………………………………. page 3

Details on the DynReaxMas procedure …………………………… page 4

Force field parameters ……………………………………………... page 5

PoLA analysis of the carbon model dataset ………………………. page 6

Comparison of Porous Volume profiles with different methods …… page 15

Comparison of volumes with different block size …………………… page 16

Mean and maximum errors for different ML features …………….. page 19

Prediction of N_2 adsorption isotherms in the model dataset ……….. page 20

¶

1) Detailed analysis of regular pores.

Even in pores of regular shape, like spheres or cylinders, not all the void has the same nature, as for the distance from walls and hence the interaction potential felt by adsorbate molecules. Unlike methods based on regular pores, which would describe such a cavity with a single "size" attributing the same nature to the whole volume, PoLA analyses the void point by point.

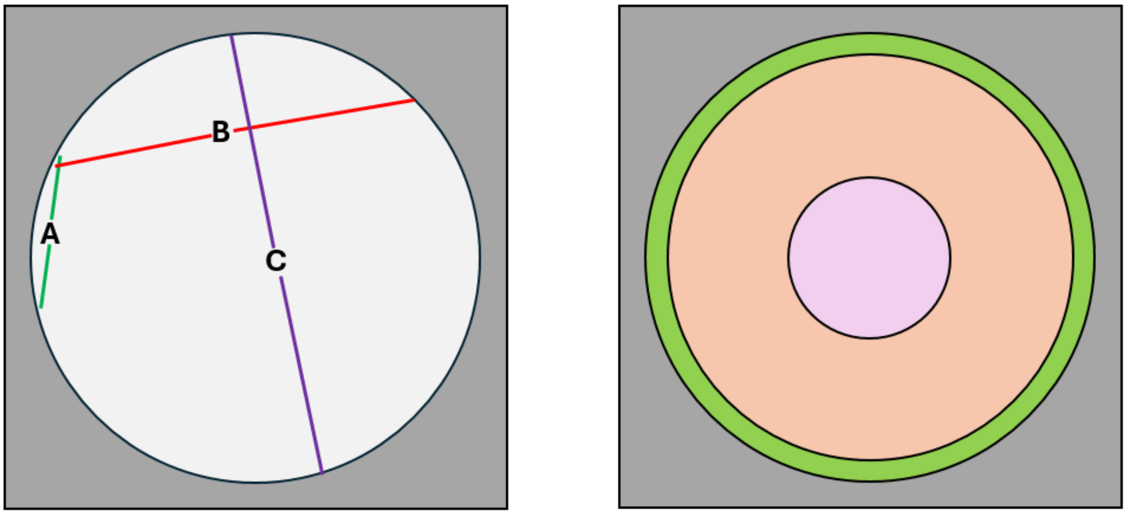

Figure S1. A spherical cavity contains points with different distances from walls. Left side: point A has a distance from opposite walls lower that 7 A, point B between 7 and 20 A, point C greater than 20 A. Right side: the green region collects all the points with distance from opposite walls lower than 7 A (which are ultra-microporous in nature, according to the expected shape of the potential), the pink and purple regions collect the microporous and the mesoporous points, respectively.

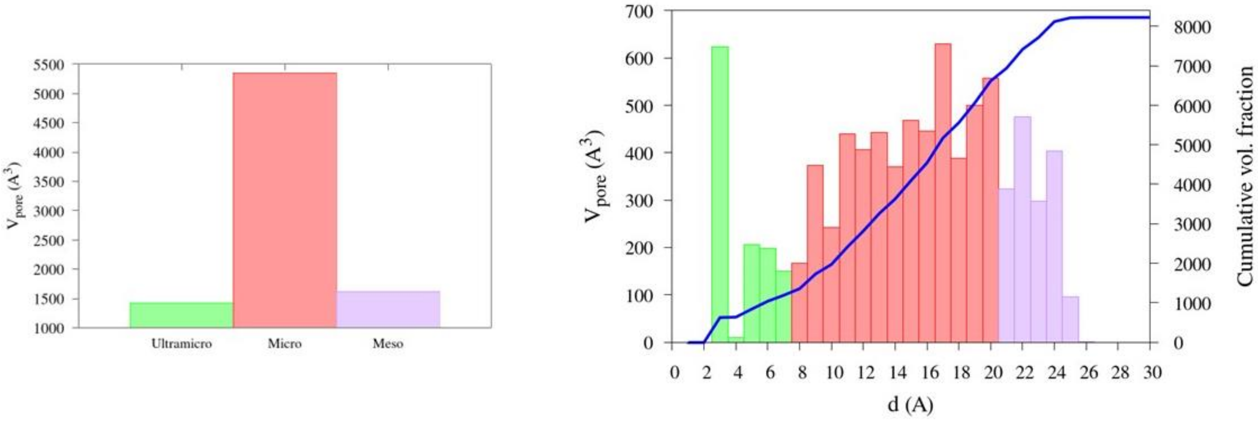

Figure S2. PoLA analysis of a spherical cavity with 25 A diameter. Left side: sum of the ultra-microporous (distance from opposite wall lower than 7 A), microporous (distance between 7 and 20 Å) and mesoporous (distance greater than 20 Å) volumes (Å^3). Right side: detailed analysis of the local porosity with 1 Å step; green, ultra-microporous; red, microporous; purple, mesoporous volumes; in blue the cumulative porous volume.

It is instructive to compare PoLA results for two systems with the same porous volumes deriving from different geometrical shapes: let's consider a solid cube of 100 Å edge, with a 2x10^5 Å ^{3} cavity obtained either as a slit pore with faces 20 Å apart, or as a collection of 48 spheres with 20 Å diameter. As shown in Figure S3, PoLA returns very different profiles for the porous volume distributions, unlike for instance PoreBlazer which would return a single peak at 20 Å for both cases.

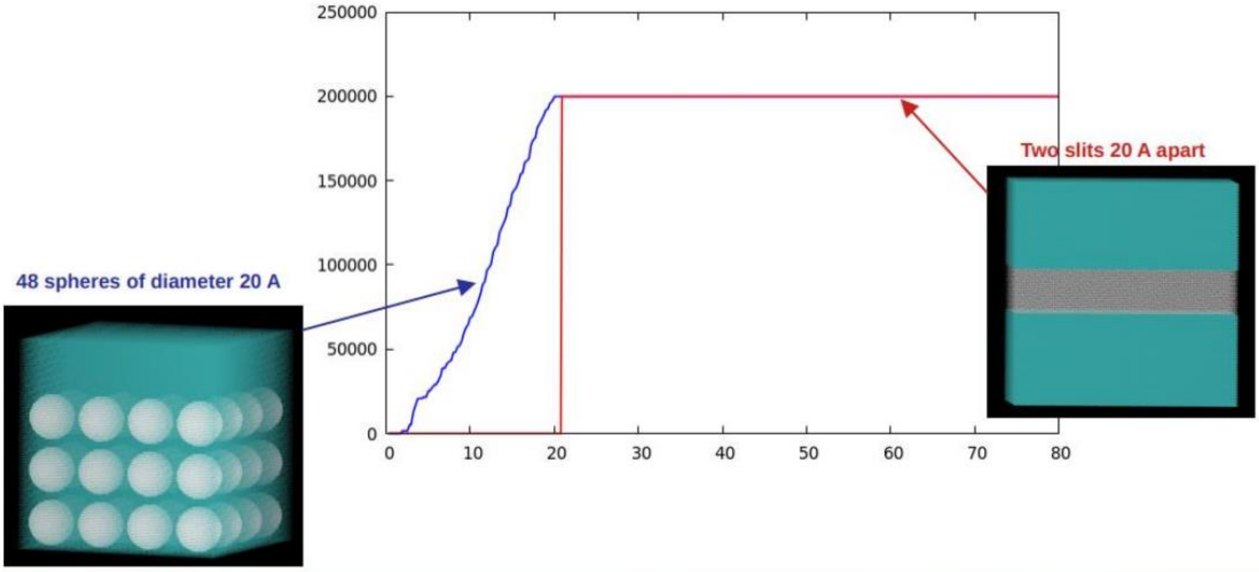

Figure S3. PoLA cumulative porous volume for two systems of 2x10^5 Å ^{3} total volume, deriving either from 20 Å diameter spheres, or from a single slit pore with faces 20 Å apart.

2) Details on the DynReaxMas procedure.

As written in the main text, some of the carbon models (i.e. structures numbered 34 to 37) were defined with the DynReaxMas protocol.

In this method, a series of R-MD simulations is first performed using the C-2013 ReaxFF force-field [10.1021/jp510274e, 10.1063/5.00528 70], with "massaged" parameter to enforce PES deformations that accelerate the breaking and reforming of the strong C-C bonds, while working at lower temperatures that are close to the experimental ones, thus achieving a more realistic process than other R-MD techniques [10.1021/acsnano.0c08029, 10.1021/acsami.4c06527].

An initial random configuration of about 5200 atoms was chosen to target a density of 0.83 g/cm^3 for models 34 to 36, well inside the range of the typical low-density carbonaceous materials. Here, DynReaxMas was applied at T = 2500 K using three different PES deformation schemes: M18, M26 and M34 (for clarity: these were named MM1/MM8, MM2/MM6 and MM3/MM4 in Ref. [10.1021/acsnano.0c08029]) to yield three pure-carbon configurations. We then applied the thermal annealing protocol of Ref. [10.1021/acsami.4c06527] ("curing") to get rid of high-energy defects.

Next, we functionalized the unsaturated carbon atoms with hydrogen atoms via the following topological/geometric protocol (in this case we did not use the reactive functionalization procedure of Ref. [10.1021/acsami.4c06527]). In detail, we deemed a carbon atom C_u to be unsaturated if bonded to only two carbon atoms (C_1 and C_2) with a relative angle between the bonds of less than

160°. In such cases, an H atom was added along the –(C_uC_1 + C_uC_2) vector at a bond distance of 1.09 Å. To conclude the structure generation procedure, a three-step protocol encompassing a minimization, a 50 ps Molecular Dynamics at T=300K, and a final minimization all using the CHONSi.ff potential [10.1021/acs.jpcc.0c01645] were conducted to equilibrate and ensure stability of the structure.

As for the larger model (number 37), massaged water probe molecules were allowed to react with the carbon surface leading to OH_2-functionalized structures bound to the under-coordinated sites. These OH_2- functionalized phases were then converted into pure C/H materials by replacing the monovalent -OH_2 groups with -H atoms. Then, the resulting models underwent the same minimization/MD/minimization equilibration scheme presented in the previous paragraph. The topological/geometric functionalization approach described above was finally performed to saturate the few residual dangling bonds with –H atoms.

3) Force field parameters.

As stated in the text, only non-bonding terms (Lennard-Jones, LJ, and electrostatic) were included in the calculation of interaction energies; the LJ parameters and the partial charges are taken from ref. 1 for N_2 and 2 for C, H, and listed in Table S1.

Table S1. LJ parameters and partial charges used in Cassandra simulations of adsorption isotherms.

**Atom type**	**σ (Å)**	**ε / k_B (K)**	**charge (e)**
C (aromatic)	3.40	28	0.000
C (aromatic, C-H)	3.40	28	-0.160
H (H-C)	2.40	12	0.160
N (N_2)	3.31	36	-0.482
D (N_2) [(a)]	0.00	0	0.964

(a) D stands for the fictitious charge in the mid of N-N bond.

1) Potoff JJ, Siepmann JI. *Vapor–liquid equilibria of mixtures containing alkanes, carbon dioxide, and nitrogen*. AIChE Journal **2001**; 47:1676-82.
2) Di Biase E, Sarkisov L. *Systematic development of predictive molecular models of high surface area activated carbons for adsorption applications*, Carbon **2013**; 64:262-28

4) PoLA analysis of the carbon model dataset.

Here follows the analysis of the porous volume in the whole model dataset used to train the machine learning algorithm.

Below, the porous volume distribution (PVD) as a function of the distance from walls.

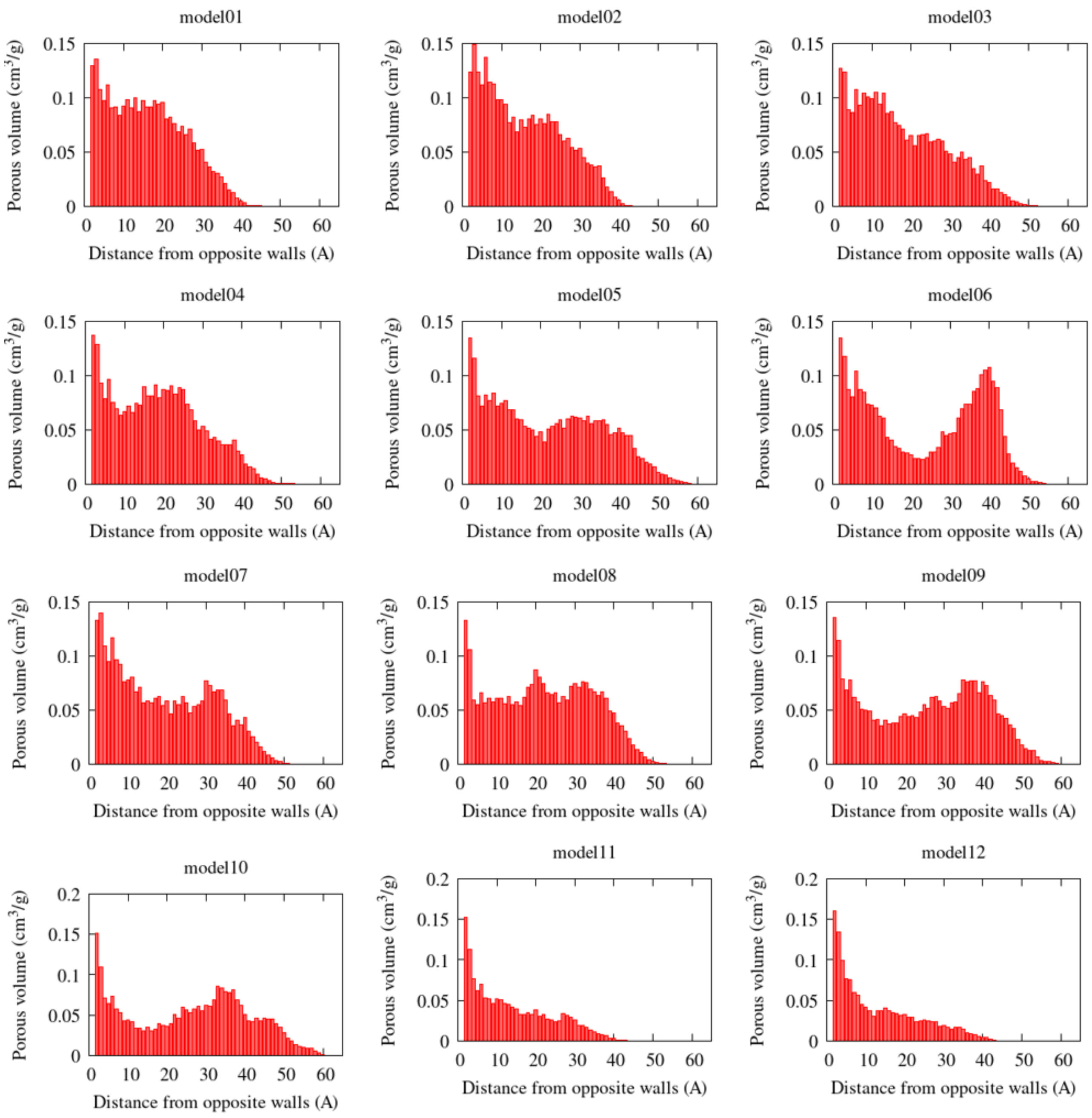

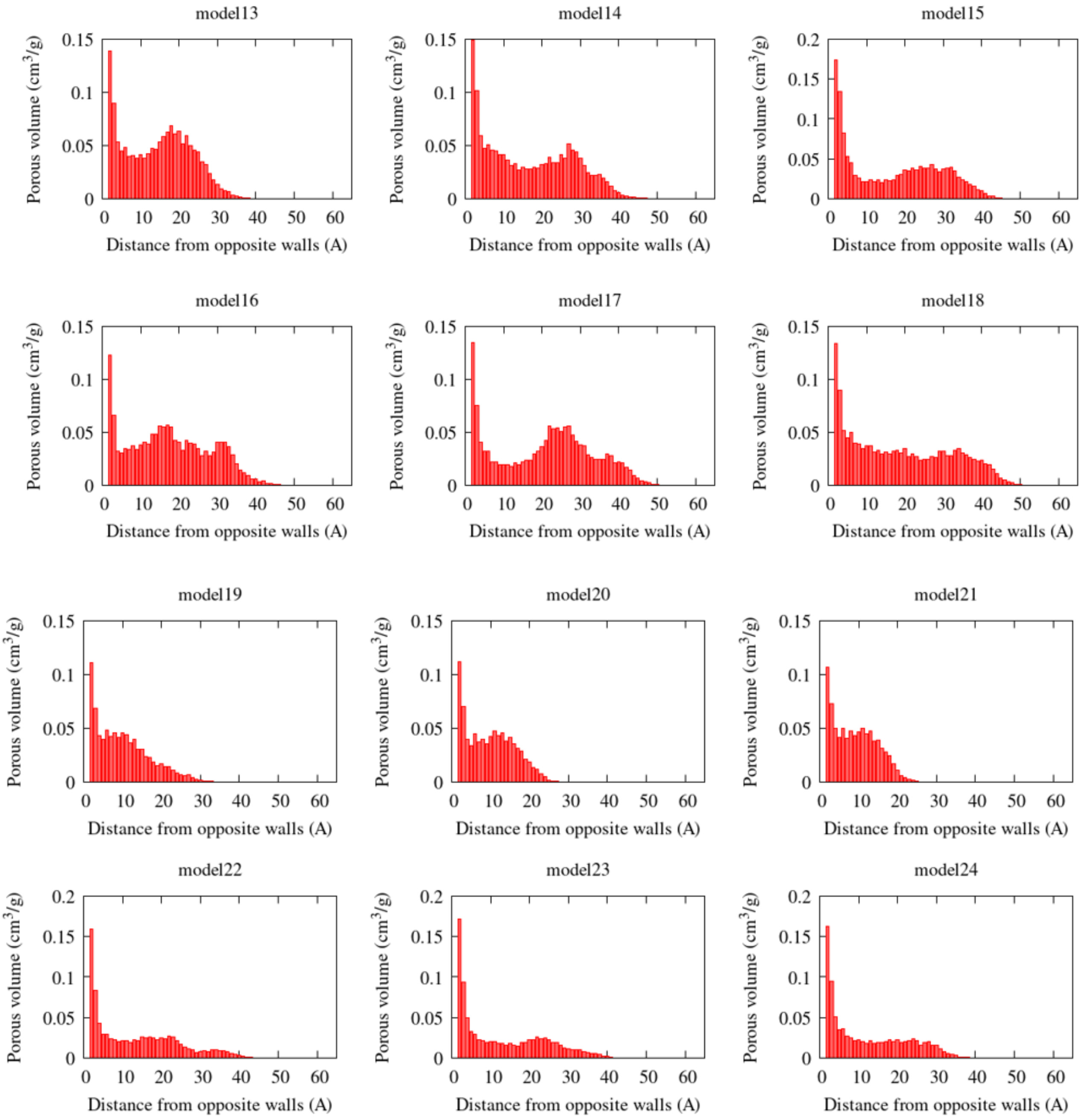
model13
model14
model15
model16
model17
model18
model19
model20
model21
model22
model23
model24
Porous volume (cm^3/g)
Distance from opposite walls (A)

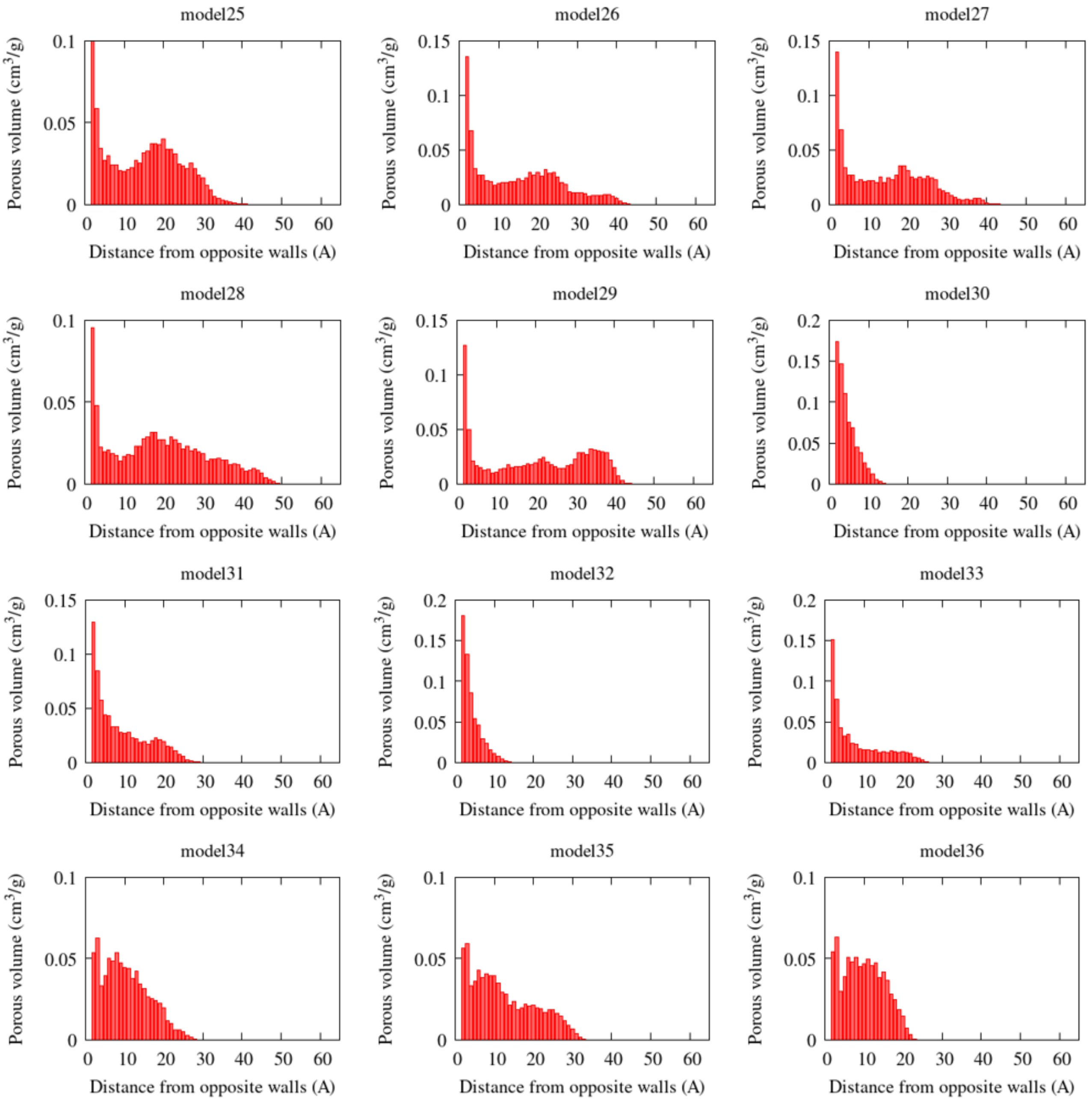
model25
model26
model27
model28
model29
model30
model31
model32
model33
model34
model35
model36
Porous volume (cm^3/g)
Distance from opposite walls (A)
0
0.05
0.1
0.15
0.2
10
20
30
40
50
60

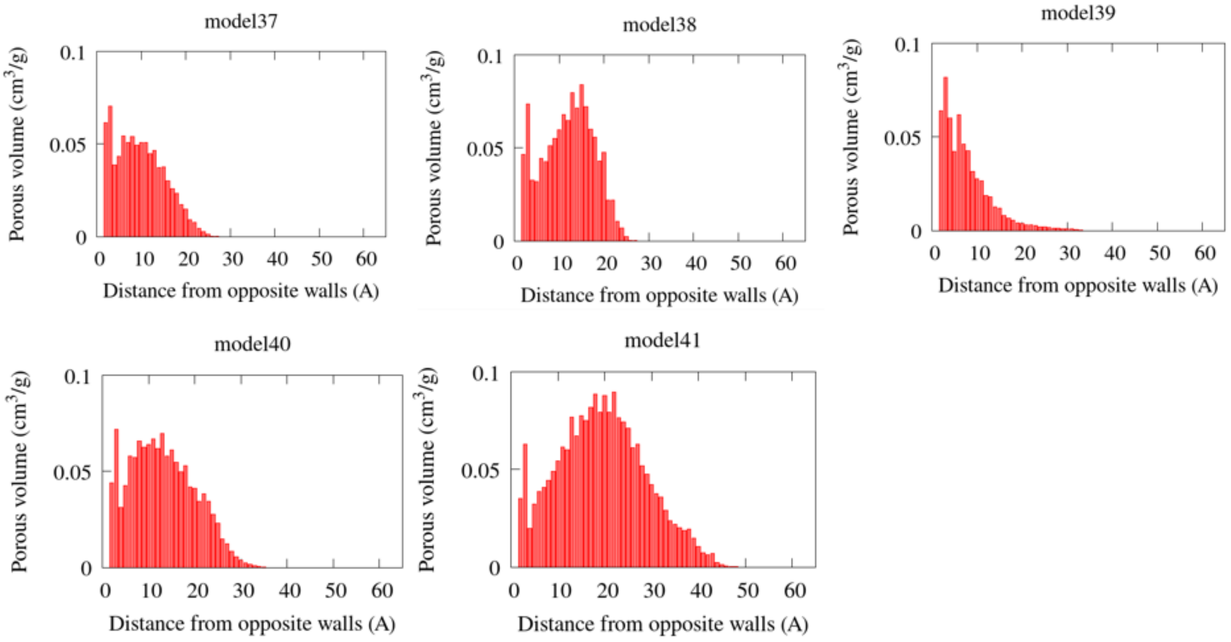

Figure S4. Porous volume distribution (PVD, cm^3/g) as a function of the minimum distance from opposite walls for all the carbon models included in the training and validation sets.

Here below, the cumulative porous volume.

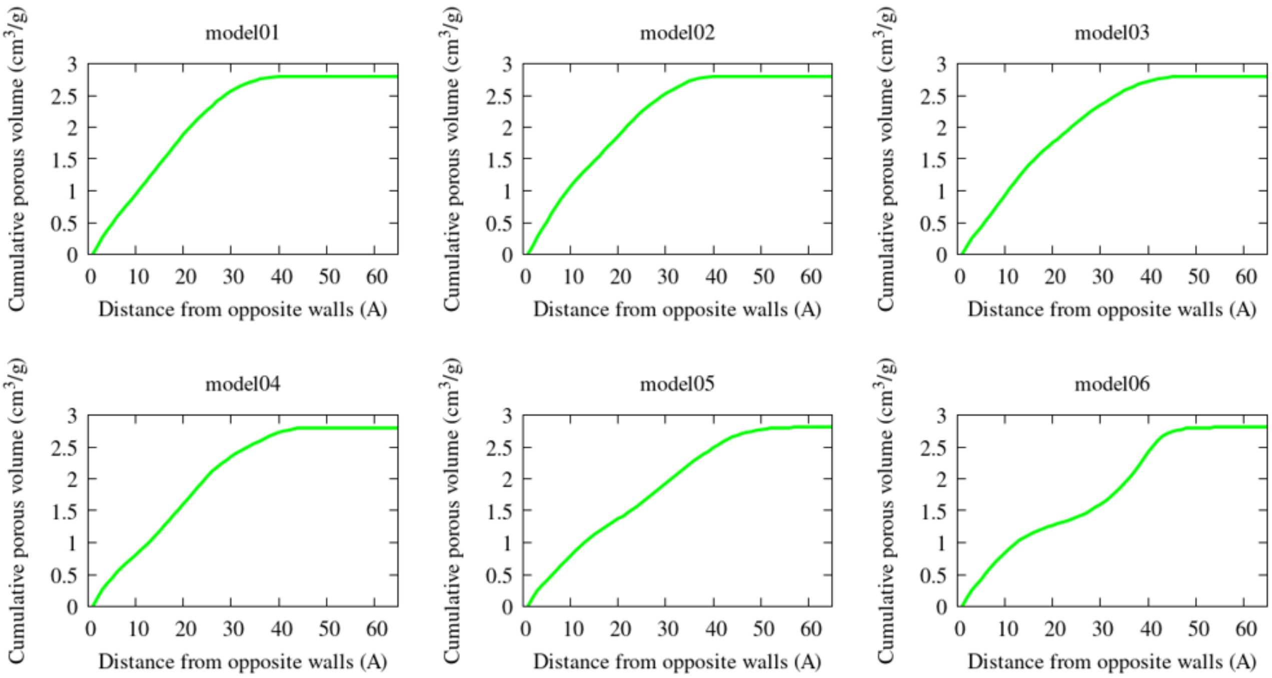

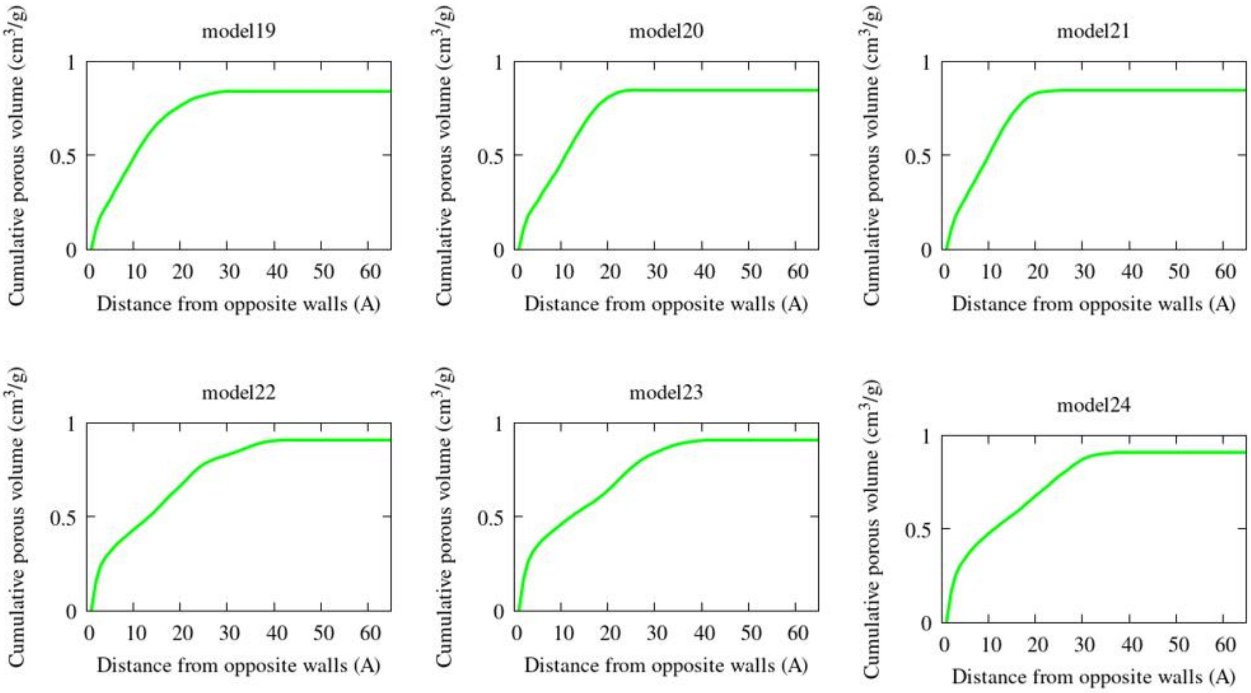
model19
model20
model21
model22
model23
model24
Cumulative porous volume (cm3/g)
Distance from opposite walls (A)
0
0.5
1
0 10 20 30 40 50 60

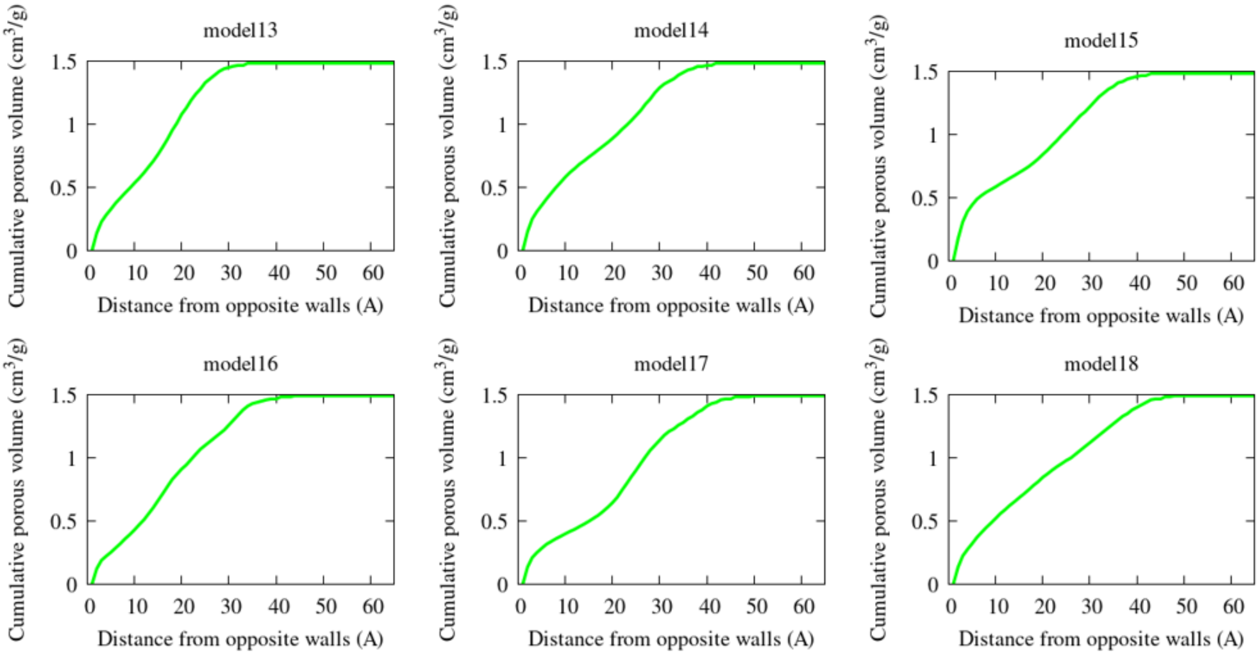
model13
model14
model15
model16
model17
model18
Cumulative porous volume (cm3/g)
Distance from opposite walls (A)
0
0.5
1
1.5
0 10 20 30 40 50 60

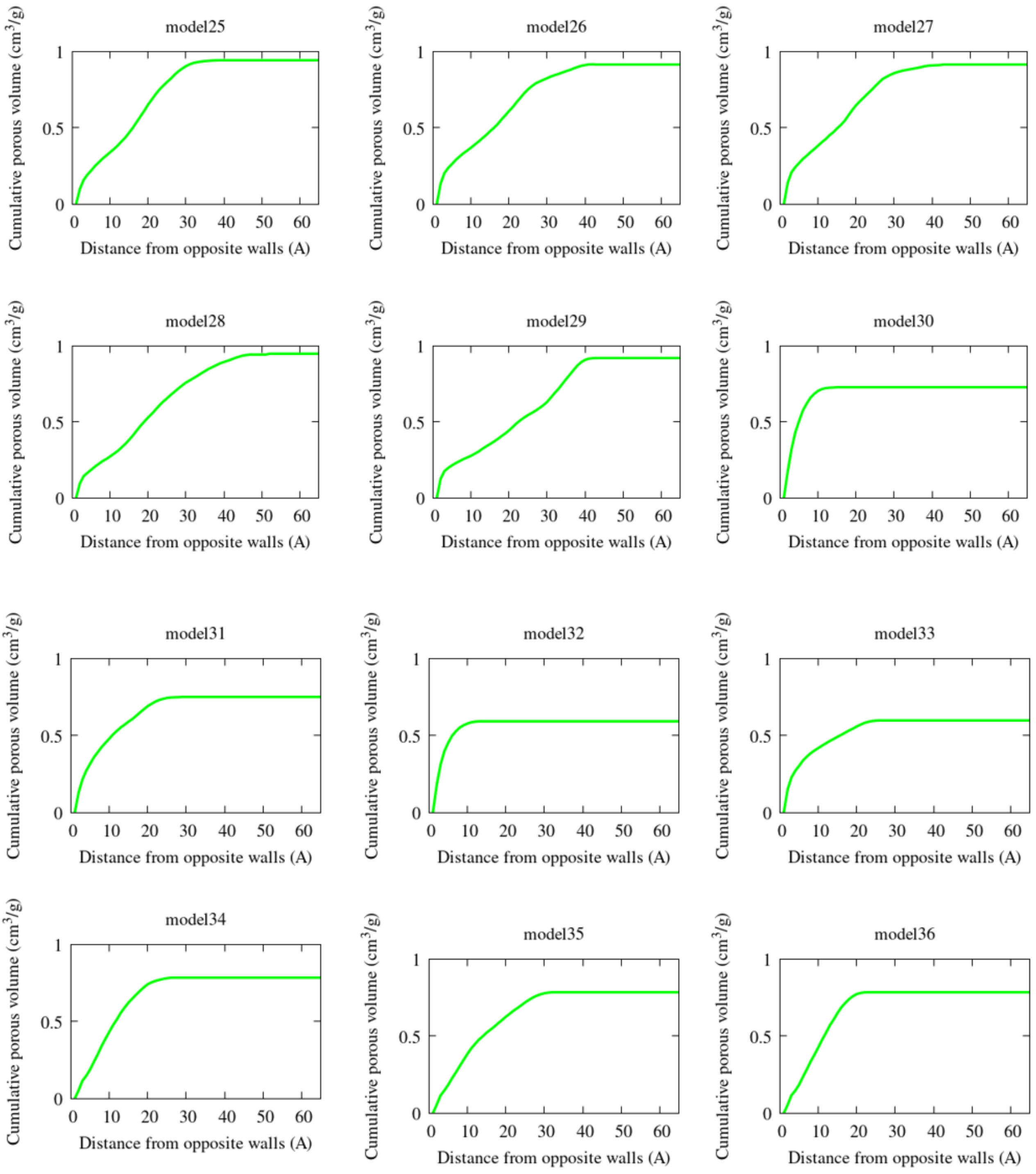
model25
model26
model27
model28
model29
model30
model31
model32
model33
model34
model35
model36
Cumulative porous volume (cm^3/g)
Distance from opposite walls (A)
0
0.5
1
0 10 20 30 40 50 60

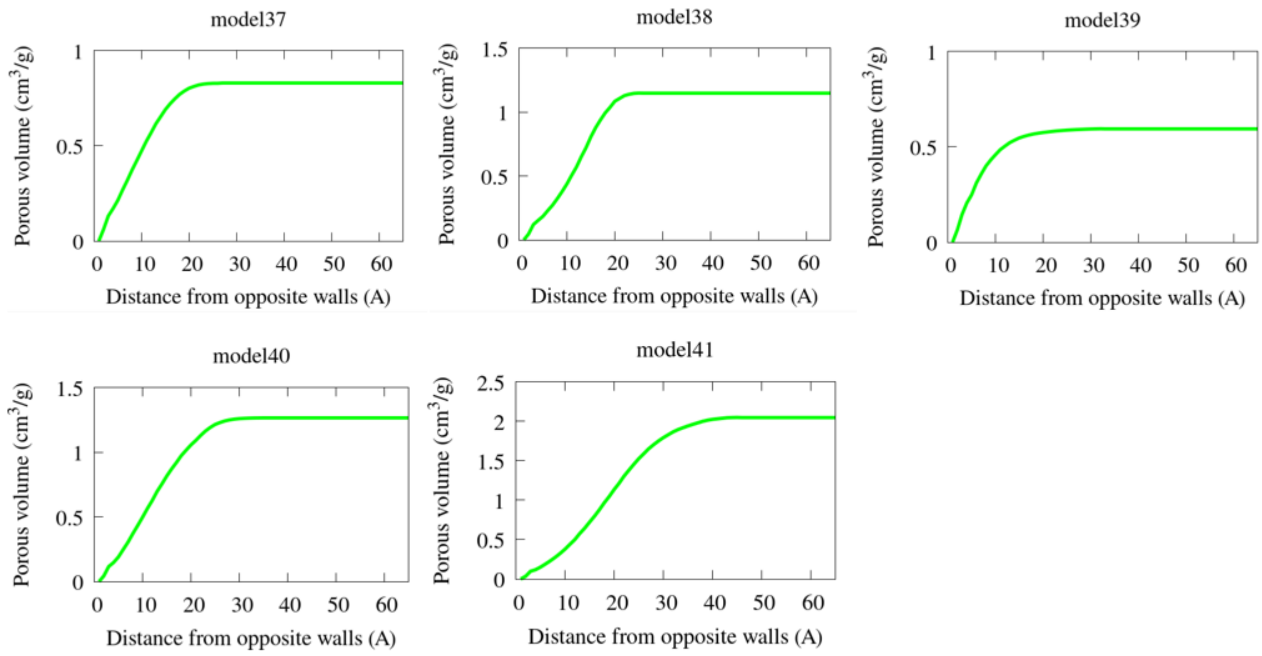

Figure S5. Cumulative porous volume (cm^3/g), i.e. the sum of the volumes reported in Fig. S4 up to various values of the distance from opposite walls, for all the carbon models included in the training set.

Table S2. Textural properties of the set of carbons used in the work, resulting from PoLA analysis. Density in g/cm^3, volumes in cm^3/g.

Model #	Density	Total porous volume	Ultra microporous volume	Microporous volume	Small Mesoporous volume	Large Mesoporous volume
1	0.300	2.803	0.674	1.214	0.848	0.068
2	0.300	2.802	0.760	1.105	0.861	0.076
3	0.300	2.803	0.626	1.130	0.819	0.226
4	0.300	2.805	0.609	0.996	0.951	0.246
5	0.300	2.806	0.564	0.812	0.851	0.544
6	0.300	2.806	0.613	0.662	0.654	0.871
7	0.300	2.803	0.691	0.874	0.907	0.329
8	0.300	2.812	0.476	0.821	1.037	0.473
9	0.300	2.810	0.537	0.571	0.834	0.811
10	0.300	2.798	0.526	0.491	0.919	0.758
11	0.500	1.476	0.529	0.548	0.367	0.033
12	0.500	1.474	0.607	0.500	0.322	0.045
13	0.500	1.482	0.416	0.661	0.402	0.003
14	0.500	1.482	0.454	0.434	0.521	0.073
15	0.500	1.479	0.518	0.324	0.543	0.094
16	0.500	1.488	0.321	0.591	0.516	0.060
17	0.500	1.486	0.338	0.307	0.636	0.204
18	0.500	1.488	0.410	0.436	0.429	0.212
19	0.748	0.844	0.354	0.411	0.080	0.000
20	0.748	0.850	0.338	0.471	0.041	0.000
21	0.748	0.847	0.361	0.469	0.016	0.000
22	0.700	0.911	0.368	0.298	0.210	0.035
23	0.700	0.908	0.400	0.242	0.245	0.021

24	0.700	0.908	0.407	0.271	0.226	0.004
25	0.700	0.946	0.275	0.378	0.285	0.008
26	0.700	0.918	0.313	0.298	0.260	0.047
27	0.700	0.914	0.318	0.331	0.239	0.025
28	0.700	0.947	0.225	0.305	0.305	0.111
29	0.700	0.922	0.243	0.202	0.337	0.140
30	0.798	0.728	0.621	0.107	0.000	0.000
31	0.798	0.751	0.393	0.299	0.059	0.000
32	0.898	0.594	0.529	0.065	0.000	0.000
33	0.898	0.599	0.364	0.196	0.040	0.000
34	0.831	0.787	0.288	0.455	0.044	0.000
35	0.831	0.785	0.266	0.360	0.159	0.000
36	0.831	0.786	0.285	0.489	0.011	0.000
37	0.792	0.828	0.319	0.483	0.026	0.000
38	0.633	1.148	0.272	0.813	0.064	0.000
39	0.990	0.595	0.356	0.219	0.020	0.000
40	0.587	1.265	0.305	0.751	0.209	0.000
41	0.404	2.049	0.230	0.904	0.806	0.110

6) Comparison of Porous Volume profiles with different methods

In the text we have compared PoLA porous volume distributions with PoreBlazer (PB) pore size distributions (PSD) for some representative models. Here below the same comparison is extended to the PSD provided by 3D-Vis and Zeo++ methods.

Model 06

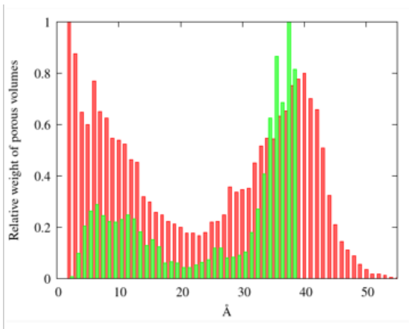

PoreBlazer

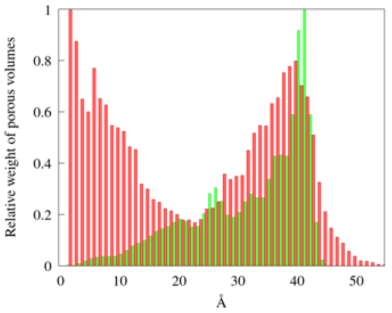

3D-Vis

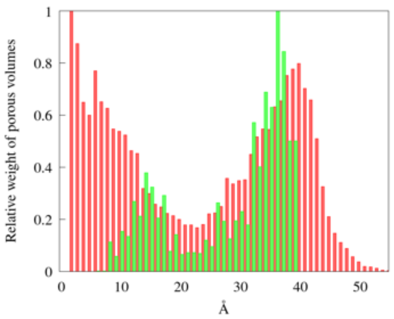

Zeo++

Model 10

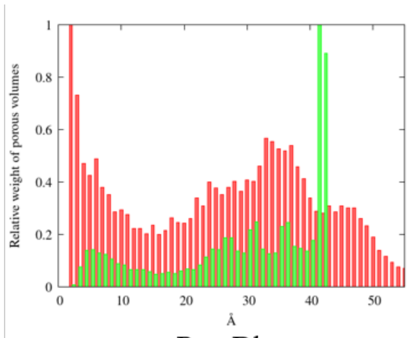

PoreBlazer

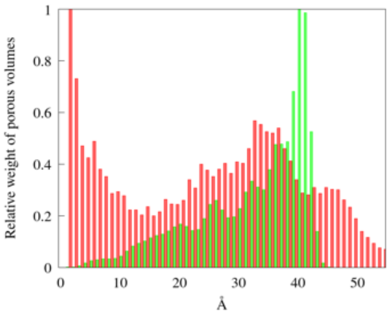

3D-Vis

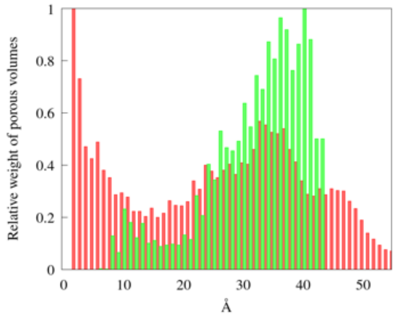

Zeo++

Model 17

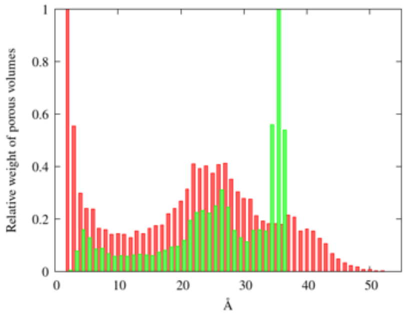

PoreBlazer

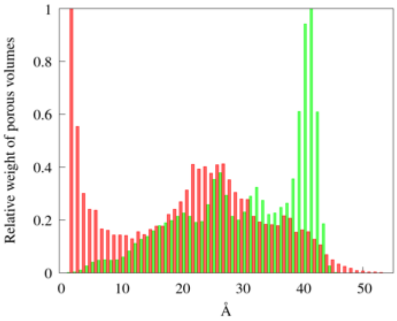

3D-Vis

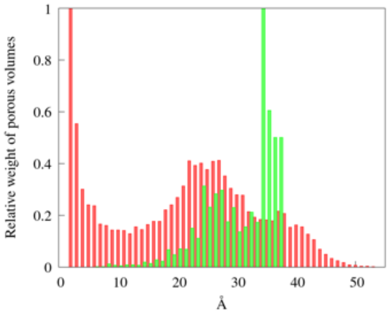

Zeo++

Model 31

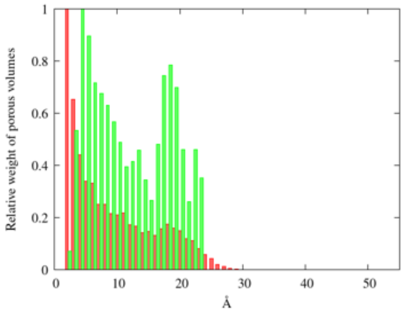

PoreBlazer

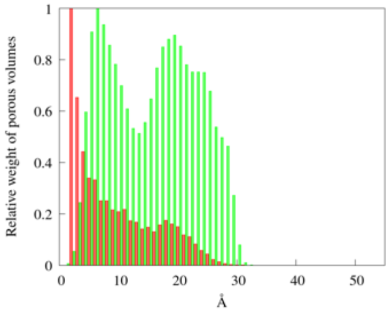

3D-Vis

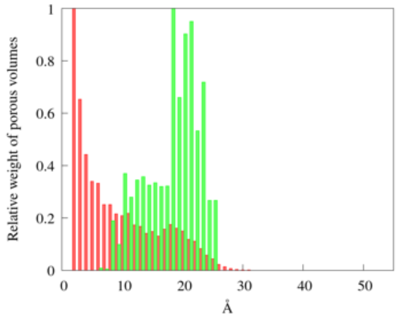

Zeo++

Figure S6. Relative Pore Size Distributions (green) provided by PoreBlazer, 3D-Vis and Zeo++ for some representative models, compared to PoLA Porous Volume Distribution (red).

Both 3D-Vis and Zeo++ share with PB the use of spherical probes, so the same considerations made in the text to comment the difference with PoLA distributions hold in these cases. 3D-Vis profiles are quite similar to PB ones, considering that the former are averaged on a number of carbon models; Zeo++ is based on a different partition of the void volume, and the resulting PSD reflect this difference.

7) Comparison of cumulative volumes with different block sizes

As stated in the text, the accuracy of the porous volume description is expected to depend on the mesh of the volume discretization, i.e. on the size of the blocks in which the cell is divided.

Here we report the cumulative porous volume as a function of the distance from opposite walls computed by PoLA for all the model dataset with block size of 1, 0.5, 0.25 Å. One can see that the three block sizes provide almost undistinguishable volumes, so 1 Å appears a safe choice to get fast and reliable results.

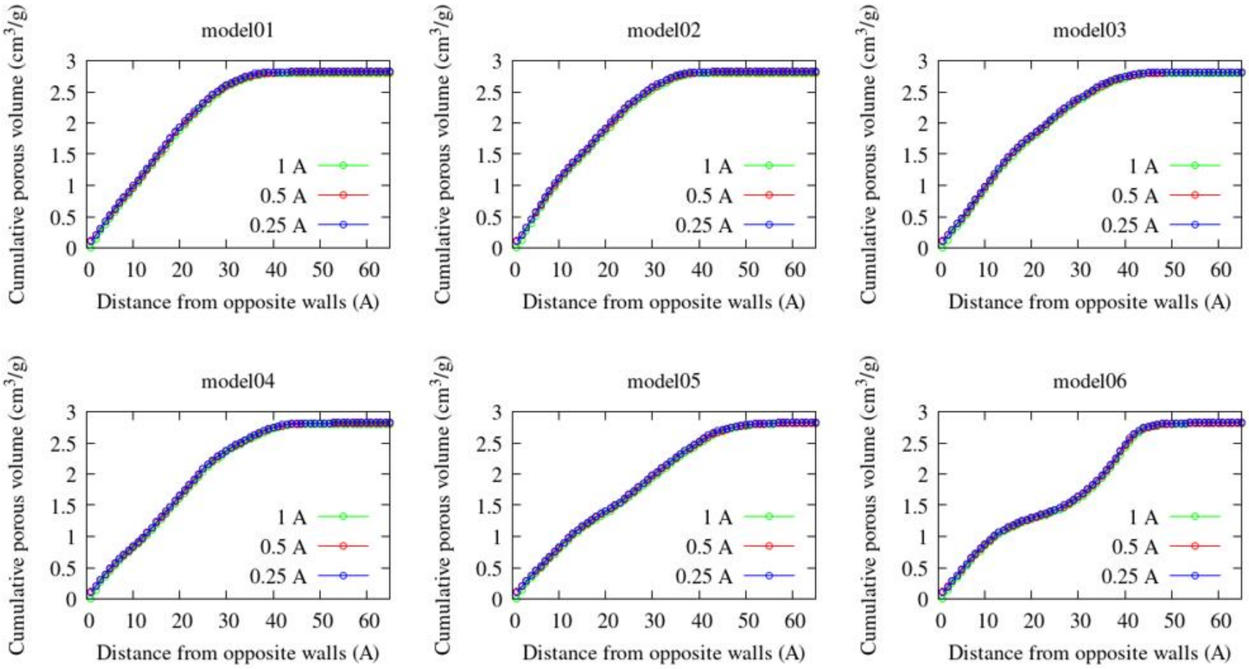

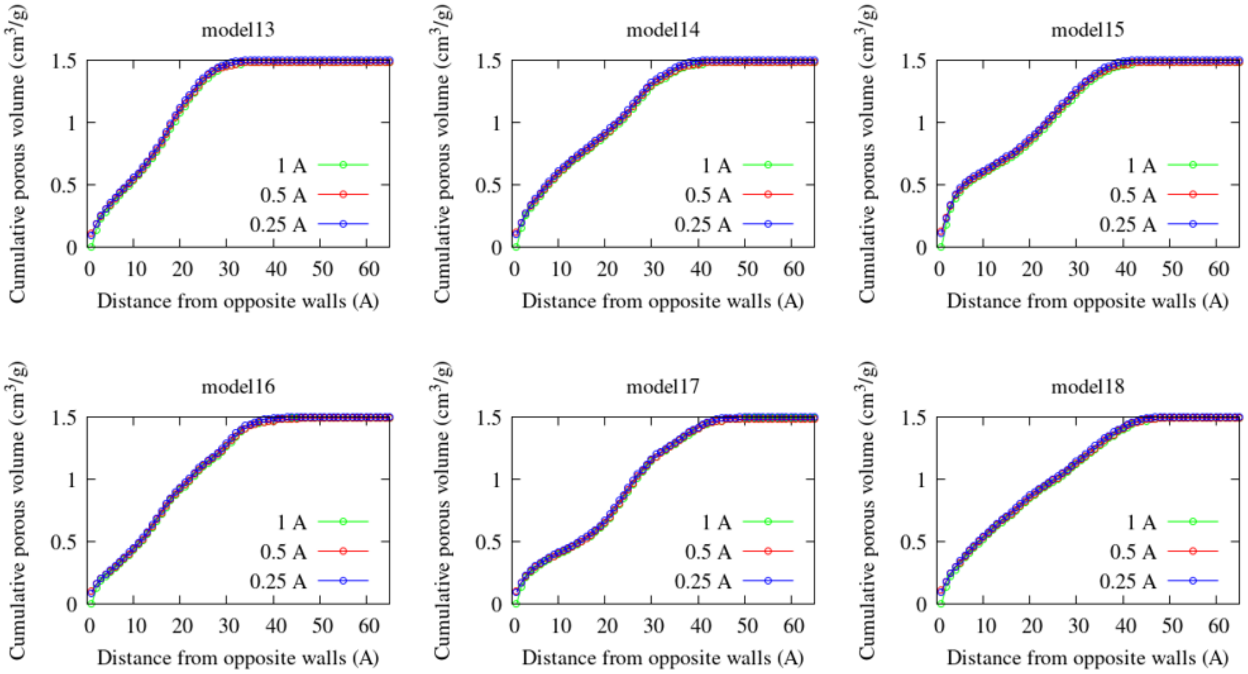
model13
model14
model15
model16
model17
model18
Cumulative porous volume (cm3/g)
Distance from opposite walls (A)
1 A
0.5 A
0.25 A
0 0.5 1 1.5
0 10 20 30 40 50 60

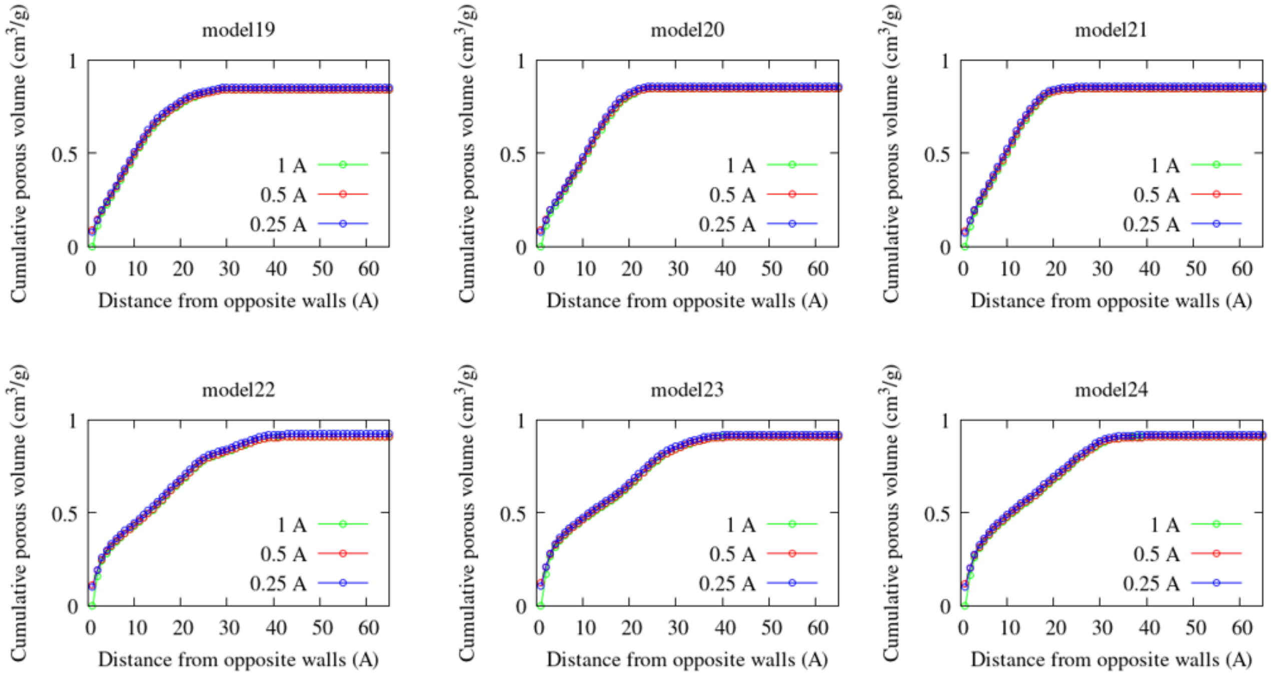
model19
model20
model21
model22
model23
model24
Cumulative porous volume (cm3/g)
Distance from opposite walls (A)
1 A
0.5 A
0.25 A
0 0.5 1
0 10 20 30 40 50 60

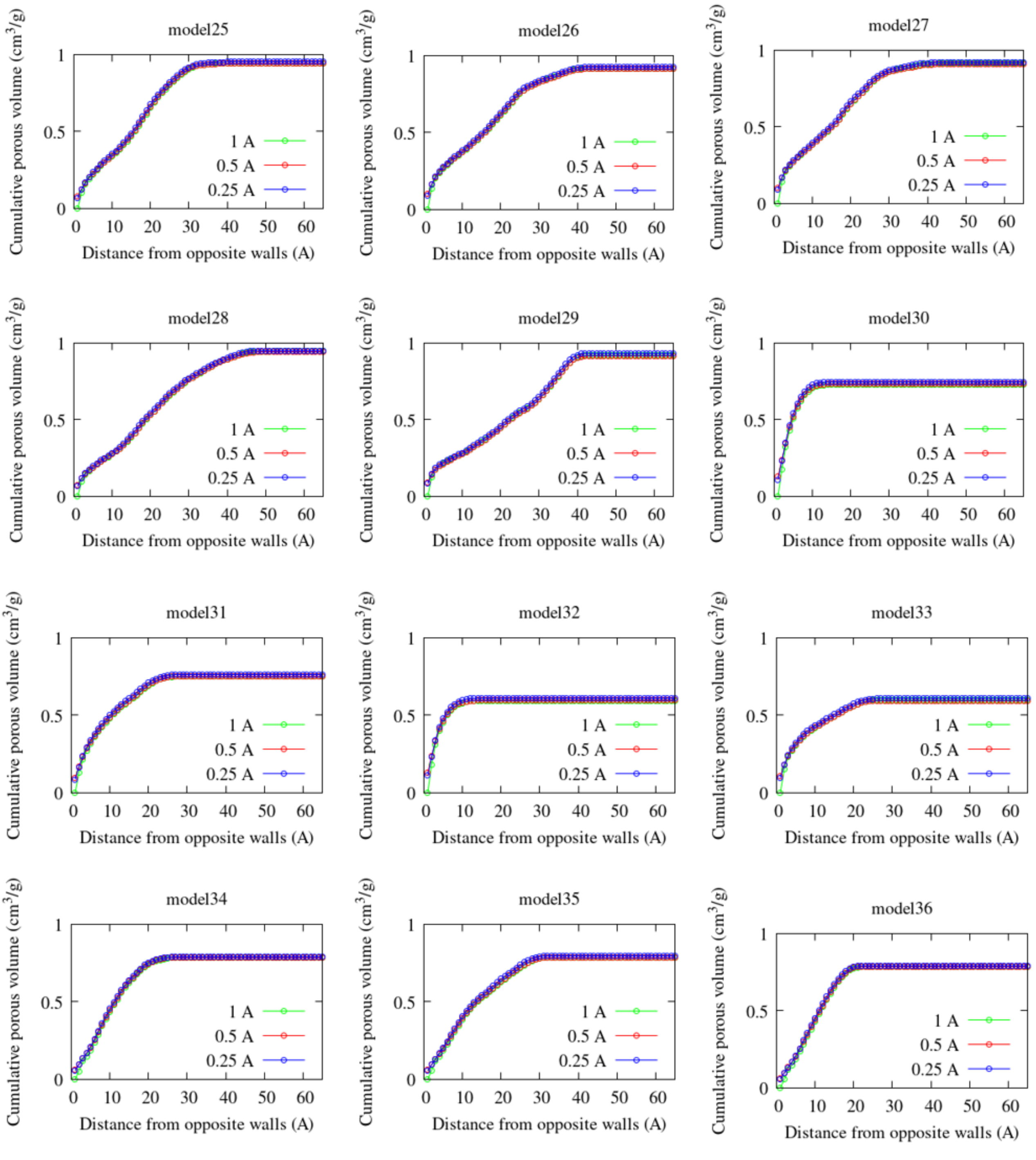

Figure S7. Cumulative porous volume (cm^3/g) for all the carbon models computed by PoLA with block size of 1, 0.5 and 0.25 A.

8) Mean and maximum errors for different ML features.

As stated in the main text, the machine learning (ML) algorithm used to correlate PoLA porous volume distributions with gas adsorption isotherms can be trained with different choices of features (i.e. the input variables used to predict the adsorption).

Indeed, we can use the porous volume distribution (PVD) itself, or rather its cumulative function (see Section 3 in this file); besides, these quantities can be provided as such or scaled by the total porous volume of each model, so the ML algorithm applies to relative adsorptions instead of absolute numbers.

Then, four regressions have been tested by predicting the nitrogen adsorption in each model of our dataset (with ML trained on the remaining models) and comparing the results with the isotherm simulated by GCMC. The comparison is performed for each pressure value and then averaged, with the results shown in Figure S7.

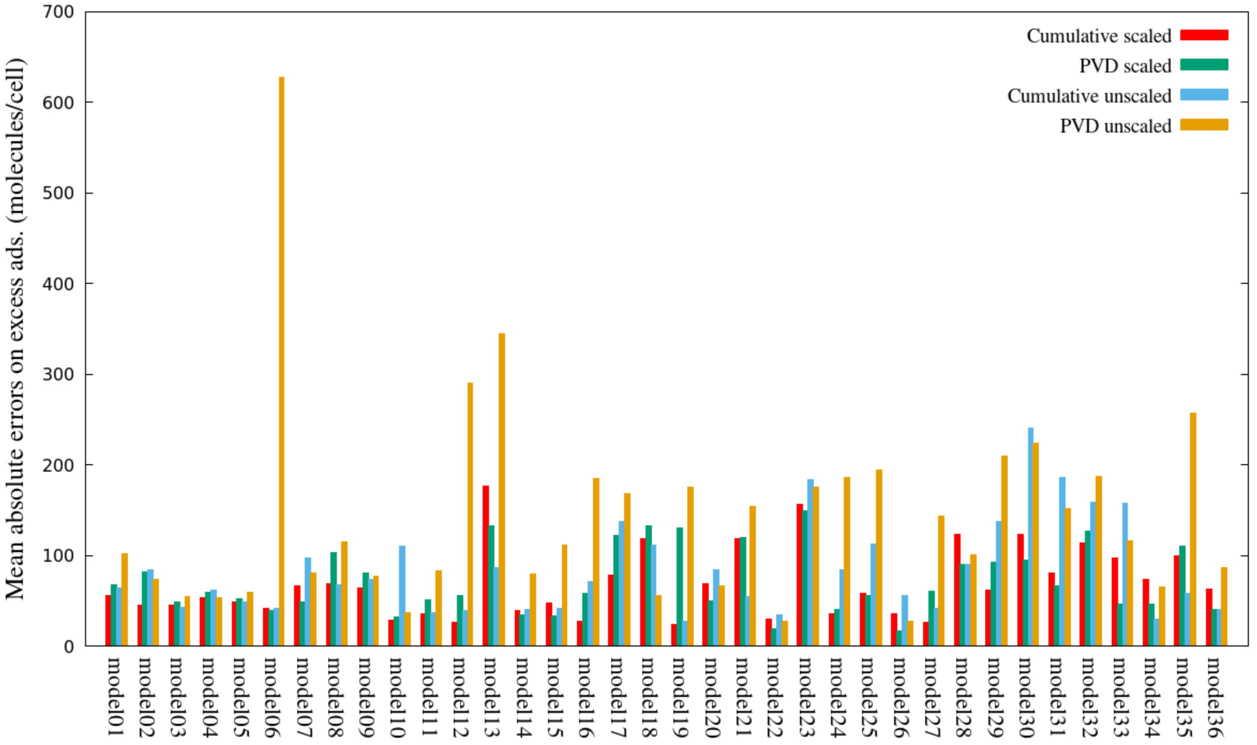

Figure S8. Mean absolute errors of predicted N_2 isotherms with respect to GCMC simulations for all the carbon models, using four different choices for ML features.

A more compact indicator is obtained with a single average of errors for all the pressure values of all the models, as reported in Table S3.

Table S3. Mean and maximum absolute errors (molecules/cell) with different choices of machine learning features.

PoLA features used for the ML training	Mean Absolute Error over the whole model dataset (molecules/cell)	Maximum Absolute Error (molecules/cell)
Cumulative volumes scaled by the total porous volumes	**68**	**176**
Unscaled cumulative volumes	**84**	**240**
PVD scaled by the total porous volumes	**72**	**150**
Unscaled PVD	**143**	**627**

9) Prediction of N_2 adsorption isotherms in the model dataset.

In the text we have shown the results of the Random Forest regression performed on the training set of 28 models, used to predict the excess isotherms on the other 8 models, collected in the test subset. The agreement was very good, indeed.

Another test can be made by selecting in turn one model as trial and using the remaining 35 as the training set: so, we can compare 36 predicted isotherms with the corresponding Monte Carlo simulations. The results are illustrated below: in this case also we find a very satisfactory agreement, confirming the reliability of the PoLA porous volumes as adsorption predictions.

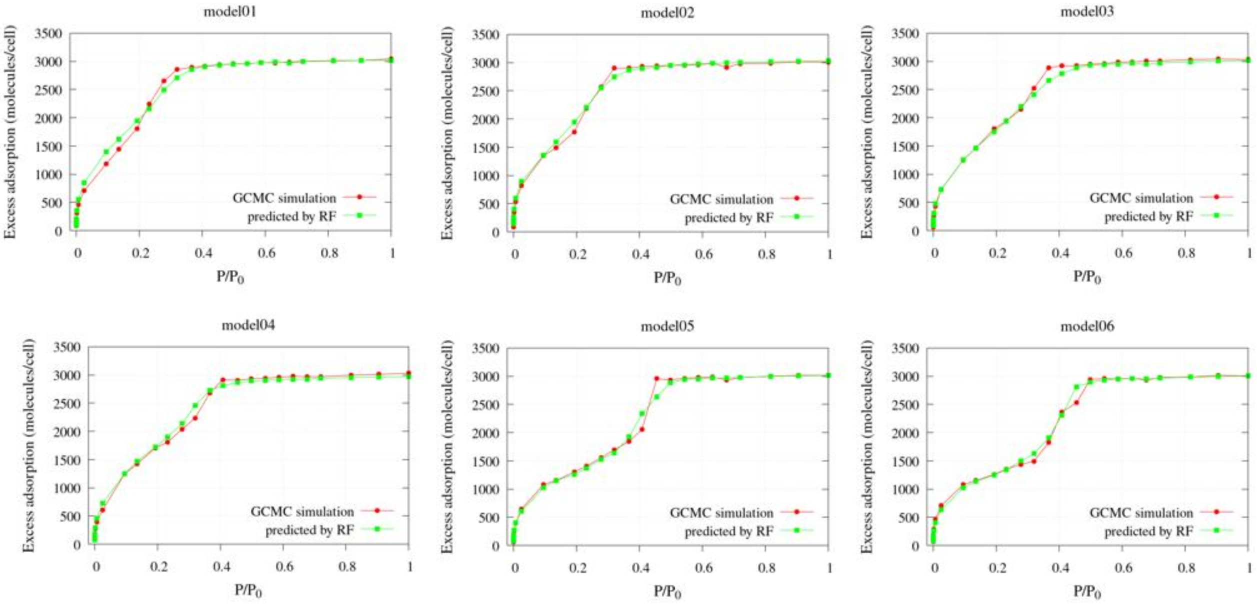

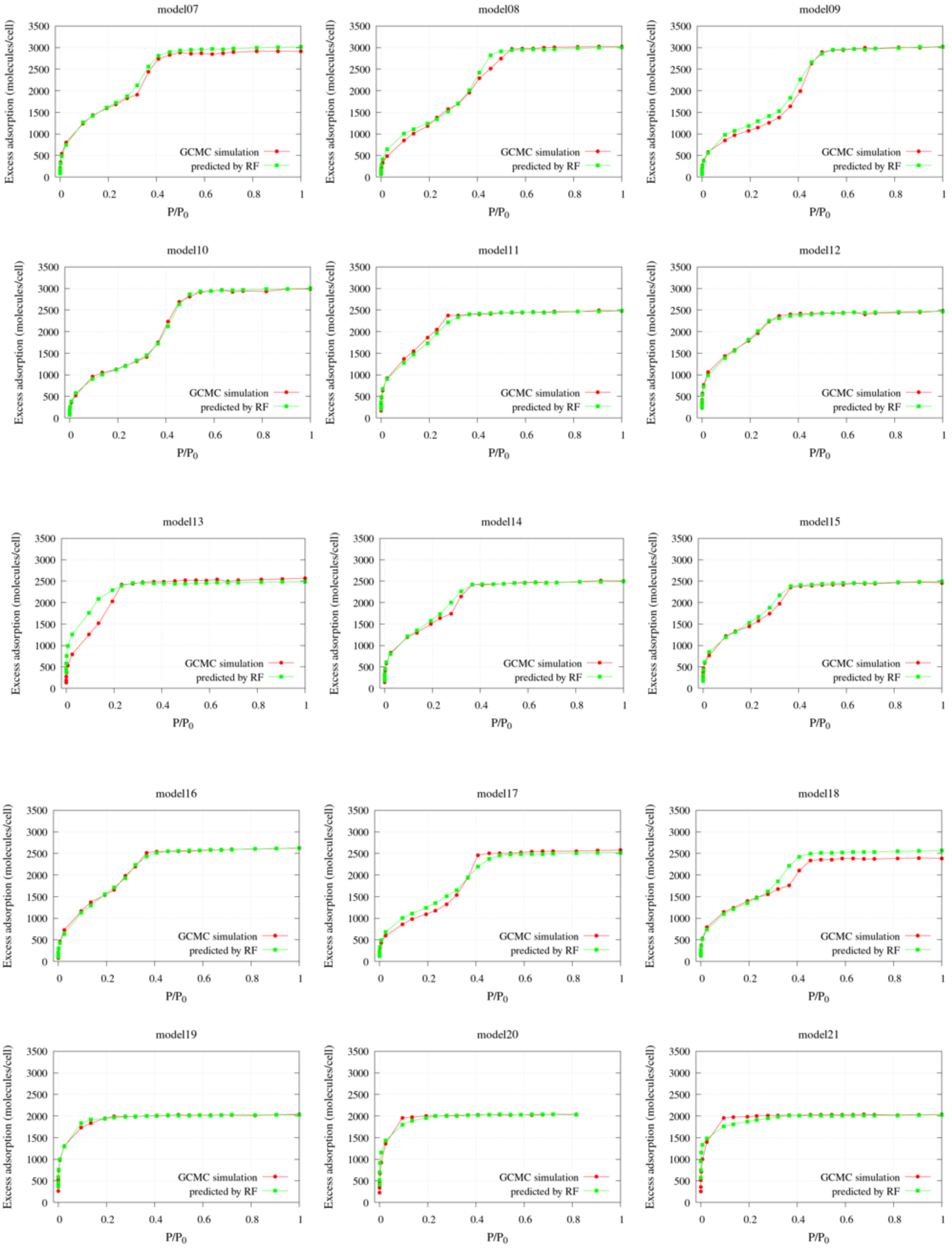
model07
model08
model09
model10
model11
model12
model13
model14
model15
model16
model17
model18
model19
model20
model21
Excess adsorption (molecules/cell)
P/P0
GCMC simulation
predicted by RF

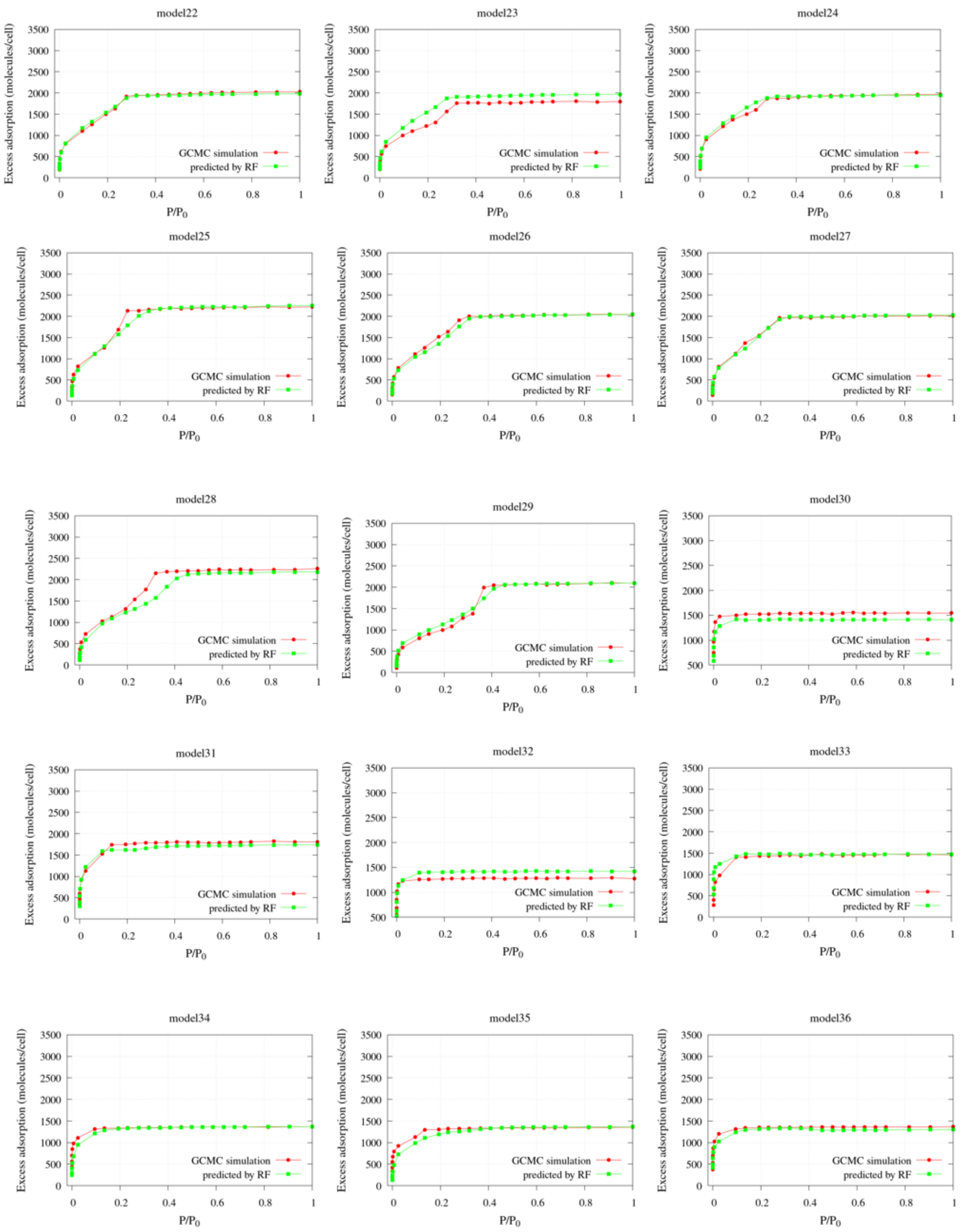

Figure S9. N_2 excess adsorption isotherms at 77 K (molecules per cell) simulated by GCMC and predicted by the Random Forest algorithm trained on the porous volumes provided by PoLA.
