# Supplementary material for: Porosity Local Analysis (PoLA): A New Approach to Describe the Porous Volume Distribution in Amorphous Carbons
Source: ACS Omega. 2025 Jul 15;10(29):31623–37. doi: 10.1021/acsomega.5c02479 (PMC12311689; doi:10.1021/acsomega.5c02479)
Supplement: Supplementary file 2 [file ao5c02479_si_002.zip › PoLA_data.zip!/graphical_abstract.pdf]

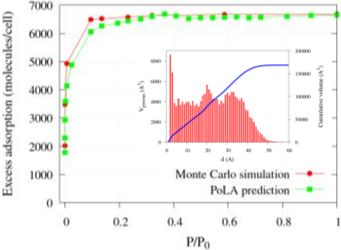

Excess adsorption (molecules/cell)
7000
6000
5000
4000
3000
2000
1000
0
0
0.2
0.4
0.6
0.8
1
P/P0
Monte Carlo simulation
PoLA prediction
